# A Non-interventional Clinical Trial Assessing Immune Responses After Radiofrequency Ablation of Liver Metastases From Colorectal Cancer

**DOI:** 10.3389/fimmu.2019.02526

**Published:** 2019-11-19

**Authors:** Markus W. Löffler, Bianca Nussbaum, Günter Jäger, Philipp S. Jurmeister, Jan Budczies, Philippe L. Pereira, Stephan Clasen, Daniel J. Kowalewski, Lena Mühlenbruch, Ingmar Königsrainer, Stefan Beckert, Ruth Ladurner, Silvia Wagner, Florian Bullinger, Thorben H. Gross, Christopher Schroeder, Bence Sipos, Alfred Königsrainer, Stefan Stevanović, Carsten Denkert, Hans-Georg Rammensee, Cécile Gouttefangeas, Sebastian P. Haen

**Affiliations:** ^1^Department of Immunology, Interfaculty Institute for Cell Biology, University of Tübingen, Tübingen, Germany; ^2^Department of General, Visceral and Transplant Surgery, University Hospital Tübingen, Tübingen, Germany; ^3^German Cancer Consortium (DKTK) and German Cancer Research Center (DKFZ) Partner Site Tübingen, Tübingen, Germany; ^4^Cluster of Excellence iFIT (EXC 2180) “Image-Guided and Functionally Instructed Tumor Therapies”, University of Tübingen, Tübingen, Germany; ^5^Department of Clinical Pharmacology, University Hospital Tübingen, Tübingen, Germany; ^6^Institute of Medical Genetics and Applied Genomics, University Hospital Tübingen, Tübingen, Germany; ^7^NGS Competence Center Tübingen (NCCT), University of Tübingen, Tübingen, Germany; ^8^Institute of Pathology, Charité – Universitätsmedizin Berlin, Berlin, Germany; ^9^Institute of Pathology, University Hospital Heidelberg, Heidelberg, Germany; ^10^Department of Diagnostic and Interventional Radiology, University Hospital Tübingen, Tübingen, Germany; ^11^Department of Radiology, Minimally Invasive Therapies and Nuclear Medicine, SLK-Hospital Heilbronn GmbH, Heilbronn, Germany; ^12^Department of Hematology, Oncology, Clinical Immunology and Rheumatology, University Hospital Tübingen, Tübingen, Germany; ^13^Department Medical Oncology and Pneumology, University Hospital Tübingen, Tübingen, Germany; ^14^Institute of Pathology and Neuropathology, University Hospital Tübingen, Tübingen, Germany; ^15^Institute of Pathology, University Hospital Marburg (UKGM) and Philipps-University Marburg, Marburg, Germany; ^16^Department of Oncology, Hematology and Bone Marrow Transplantation With Division of Pneumology, University Medical Center Hamburg-Eppendorf, Hamburg, Germany

**Keywords:** colorectal cancer, radiofrequency ablation, liver metastasis, HLA ligandome, T cells, tumor-associated antigens, neoepitopes, abscopal effect

## Abstract

**Background:** Radiofrequency ablation (RFA) is an established treatment option for malignancies located in the liver. RFA-induced irreversible coagulation necrosis leads to the release of danger signals and cellular content. Hence, RFA may constitute an endogenous *in situ* tumor vaccination, stimulating innate and adaptive immune responses, including tumor-antigen specific T cells. This may explain a phenomenon termed abscopal effect, namely tumor regression in untreated lesions evidenced after distant thermal ablation or irradiation. In this study, we therefore assessed systemic and local immune responses in individual patients treated with RFA.

**Methods:** For this prospective clinical trial, patients with liver metastasis from colorectal carcinoma (mCRC) receiving RFA and undergoing metachronous liver surgery for another lesion were recruited (*n* = 9) during a 5-year period. Tumor and non-malignant liver tissue samples from six patients were investigated by whole transcriptome sequencing and tandem-mass spectrometry, characterizing naturally presented HLA ligands. Tumor antigen-derived HLA-restricted peptides were selected by different predefined approaches. Further, candidate HLA ligands were manually curated. Peripheral blood mononuclear cells were stimulated *in vitro* with epitope candidate peptides, and functional T cell responses were assessed by intracellular cytokine staining. Immunohistochemical markers were additionally investigated in surgically resected mCRC from patients treated with (*n* = 9) or without RFA (*n* = 7).

**Results:** In all six investigated patients, either induced immune responses and/or pre-existing T cell immunity against the selected targets were observed. Multi-cytokine responses were *inter alia* directed against known tumor antigens such as cyclin D1 but also against a (predicted) mutation contained in ERBB3. Immunohistochemistry did not show a relevant influx of immune cells into distant malignant lesions after RFA treatment (*n* = 9) as compared to the surgery only mCRC group (*n* = 7).

**Conclusions:** Using an individualized approach for target selection, RFA induced and/or boosted T cell responses specific for individual tumor antigens were more frequently detectable as compared to previously published observations with well-characterized tumor antigens. However, the witnessed modest RFA-induced immunological effects alone may not be sufficient for the rejection of established tumors. Therefore, these findings warrant further clinical investigation including the assessment of RFA combination therapies e.g., with immune stimulatory agents, cancer vaccination, and/or immune checkpoint inhibitors.

## Introduction

Percutaneous radiofrequency ablation (RFA) has initially been established as a therapeutic modality enabling the physical destruction of malignant tissue by heat. During RFA, an alternating electric current is generated within the tissue leading to ion agitation and frictional heat, resulting in coagulative necrosis of cells due to local heating of tissues (>60°C) (1, 2). This minimally invasive technique is an additional therapeutic option or alternative to surgical treatment, mainly applied for patients for whom a complete surgical tumor resection cannot be achieved or who do not qualify for surgery due to other reasons.

Besides various other malignancies, RFA is frequently used to reach tumor control in colorectal cancers (CRC) metastasized to the liver (mCRC), where it has been established as a safe and effective procedure (3, 4). Since recurrence rates surpass 50% for patients undergoing potentially curative liver resection for mCRC (5), RFA may not only constitute a promising adjunct treatment approach, but also have beneficial effects beyond local tumor control. Nevertheless, the definite benefit of RFA treatment in mCRC of the liver remains to be established (6), and respective randomized controlled trials are still ongoing (7).

It has only been appreciated recently that RFA treatment may also have profound immunological implications and that there are effects occurring beyond mere local tumor destruction (8). Like radiotherapy and cryoablation, RFA may also induce so-called abscopal effects, where subsequent to the treatment of one malignant lesion, another untreated distant lesion responds to treatment. The phenomenon is still insufficiently understood (9) and even in mouse models no robust effects are observed (10). However, particularly in mouse models there is convincing evidence that the involvement of the immune system represents the most plausible mode of action, since RFA and comparable treatment approaches may constitute a form of *in situ* whole cell vaccination comparable to lysates from tumor cells. Such tumor cell lysates have been proposed to contribute a wide array of immunogens that may induce tumor rejection (11–13). Abscopal effects have been ascribed to the stimulation of tumor-specific T cells recognizing tumor antigen-derived HLA-restricted peptides. Interestingly, these effects were shown to occur with disproportionally high frequency in malignancies considered as immunogenic such as malignant melanoma, renal cell carcinoma, and lymphomas (12) but they still remain rare and cannot be regularly reproduced (14–16). In this context, it can be assumed that single T cell targets such as mutated HLA ligands bear great potential for tumor rejection and may even hold the key for patient cure, in case they can be specifically exploited for therapy (17). Nonetheless, based on the current state-of-the-art in characterizing HLA-presented ligands by mass spectrometry (MS), mutated HLA ligands are probably very rare. This aspect is of particular relevance for malignancies with very few mutations (18). Only a small fraction of predicted mutated gene products was detectable by MS on tumors (19) or shown as immunogenic and may therefore mediate tumor rejection (20), a notion that may also help to explain the sporadic nature of abscopal effects.

In CRC for instance, highly mutated (e.g., microsatellite instable) cancers were shown to respond to immune checkpoint inhibition (ICI) immunotherapies, whereas sporadic CRC with low mutation rates did not (21). Further, it is becoming clearer, that not only mutated HLA ligands may drive the immune response against cancers, but also alterations beyond exome-derived mutations may prove relevant for the rejection of malignant cells, such as (non-mutated) neoantigens, originating from tumor-specific alterations, protein modifications, RNA-editing and alterations in non-coding regions (22–25). Excluding some exceptions, most of these alterations are patient-individual. In addition, there have been recent reports that ICI in combination with radiotherapy can increase the occurrence of clinically significant abscopal effects (26).

In a previous study, we have shown that tumor antigen-specific antibodies and T cells can be induced in a fraction (<10%) of patients following RFA treatment (27). In this study, we aimed at studying patient-individual anti-tumor T cell responses occurring in the context of RFA in patients with metastasized colorectal cancer (mCRC), as well as assessing immune infiltrates that may arise in distant metastases following RFA treatment.

## Materials and Methods

### Ethics Approval and Informed Consent

This trial using the acronym IRISS (Interventional Radiology, Immunology, Surgery Study) was conducted in accordance with the principles of the Declaration of Helsinki and approved by the local institutional review board of the University Hospital Tübingen (Reference No. 169/2005V and 638/2014BO2). All participants provided written informed consent before study inclusion.

### Study Design and Patients

**Figure 1:**
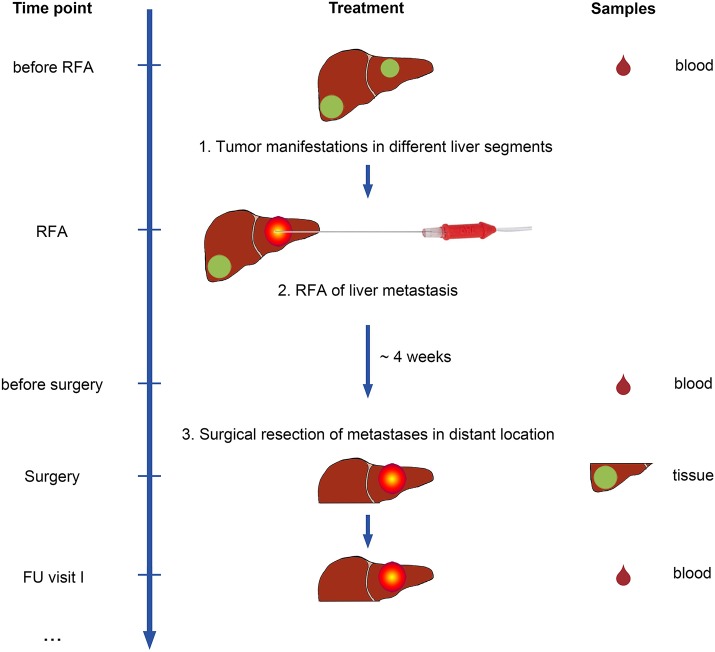
Study design of the IRISS trial. Patients with metastasized colorectal cancer (mCRC) and tumor manifestations in different liver segments were included in the study. Patients underwent RFA treatment for one malignant liver lesion first. After ~4 weeks, a second lesion was surgically removed. As a control group, mCRC patients were included who underwent surgical resection only. Blood samples (drops, right) were collected at predefined time points before initiation of treatment and during follow-up visits. Tumor and non-malignant liver (NML) tissue was obtained from surgical specimens for analysis.

Sixteen patients were recruited for this study. Patient characteristics are provided in Supplementary Table 1. The first group (Figure 1) included all consenting patients with metastases from CRC in different liver segments, scheduled for treatment with RFA and subsequent liver surgery at Tübingen University Hospital, recruited in the course of a 5-year period (*n* = 9). This RFA + surgery group [all men; mean age 64 years (range, 45–79 years) at initial diagnosis] included six patients with sufficient sample materials for in-depth analyses (mCRC and non-malignant liver (NML) tissues, as well as PBMCs for immunological evaluation). Patients in this group were treated with one session of RFA for one of the tumor lesions, followed by subsequent surgical resection (on average 4 weeks after RFA; range 1–8 weeks) of the non-RFA-pretreated, distant liver metastases.

A corresponding control group included patients (*n* = 7) with liver metastases of mCRC scheduled for surgery only (five males; mean age 58 years (range, 45–77 years) at initial diagnosis). Of note, no fresh frozen tumor/NML tissue or PBMCs was available for this group and only paraffin embedded tumor tissue was accessible for immunohistochemical evaluation.

All patients were treated with curative intent according to institutional standards and presented with a median number of two mCRC lesions (min. – max.: 1–7).

### Sample Materials

For patients included in the RFA + surgery group and evaluated in immunological experiments (*n* = 6), blood samples were collected before RFA treatment, at surgery (~1 month later), and at several follow-up visits thereafter, at intervals of 1–4 months (Figure 1). Peripheral blood mononuclear cells (PBMCs) were isolated by density gradient centrifugation and cryopreserved in freezing medium [fetal calf serum (FCS) with 10% dimethylsulfoxide (DMSO)] until subsequent analysis.

Additionally, during elective liver surgery for mCRC, scheduled after RFA treatment, resected tissue was obtained from both mCRC as well as NML tissue. Tissue samples without diagnostic relevance were divided and snap frozen in liquid nitrogen or else stored in RNA later (ThermoFisher Scientific, Waltham, MA) and kept at −80°C for long-term cryopreservation until analysis.

For all patients from both groups (*n* = 16), mCRC tissue samples were paraffin embedded, and diagnosis was confirmed by expert pathologist review. Paraffin embedded tissue was used for immunohistochemical evaluation.

### HLA Typing

For the patients included in the RFA + surgery group (*n* = 6), high-resolution HLA typing from peripheral blood (LUMINEX and sequence-based typing according to implemented validated institutional clinical routines) was performed for HLA-A and HLA-B (Table 1).

**Table 1:** Patient characteristics and results of HLA ligandomics performed by tandem mass spectrometry.

**UPN**	**Diagnosis**	HLA-A*		HLA-B*		**Tissue**	**Sample weight**	**HLA class I**			**HLA class II**	**RIN**
							**[mg]**	**Peptides (n=)**	**Binders (n=)**	**Binders [%]**	**Peptides (n=)**	
IRISS01	mCRC	24	66	27	44	Tumor	160	1,785	1,508	84.5	850	7.2
						NMT	710	1,917	1,507	78.6	1490	7.1
IRISS05	mCRC	01	02	08	18	Tumor	46	260	198	76.2	556	8.4
						NMT	290	922	820	88.9	803	7.1
IRISS06	mCRC	02	24	15	35	Tumor	54	711	666	93.7	n.d.	7.4
						NMT	n.d.	n.d.	n.d.	n.d.	n.d.	3.3
IRISS08	mCRC	02	33	14	18	Tumor	24	231	175	75.8	220	8.6
						NMT	280	1101	923	83.8	631	7.5
IRISS09	mCRC	01	08	Tumor	n.d.	n.d.	n.d.	n.d.	n.d.	n.d.
						NMT	130	560	341	60.9	445	8.3
IRISS12	mCRC	01	02	08	27	Tumor	920	1887	1714	90.8	1461	6.9
						NMT	840	1372	1244	90.7	1307	8.3

*CRC, colorectal cancer; HLA, human leukocyte antigen; m, metastasized; n.d., not determined; NMT, non-malignant tissue; RIN, RNA integrity number; RNA, ribonucleic acid; UPN, uniform patient number. Binders were defined as HLA-eluted peptides predicted to bind to the respective HLA alleles of the patient above the thresholds given in Materials and Methods determined by suitable software*.

### Isolation of HLA Ligands From Surgical Specimens

Immunoaffinity purification was used for parallel isolation of HLA class I and II molecules from tissue lysates, employing the pan HLA class I monoclonal antibody W6/32 (28) as well as the HLA-DR monoclonal antibody L243 (29) together with the pan HLA class II monoclonal antibody Tü39 (30) (all produced in-house at the Department of Immunology, University of Tübingen, Germany) as previously described (31). HLA class I and II-bound peptides were separately eluted using 0.2% trifluoroacetic acid.

### Analysis of HLA Ligands by LC-MS/MS

Purified HLA-bound peptides from HLA class I and II immunoprecipitates were analyzed in up to six technical replicates of each sample, as previously described (32). Briefly, purified peptides were separated by nanoflow ultra-high-performance liquid chromatography (uHPLC; UltiMate 3000 RSLCnano System, ThermoFisher) using a 50 μm ×25 cm column (PepMap RSLC, ThermoFisher) and an acetonitrile gradient ranging from 2.4 to 32.0% over the course of 90 min. uHPLC eluting peptides were analyzed in an online coupled linear trap quadrupole (LTQ) Orbitrap XL mass spectrometer (ThermoFisher), equipped with a nanoelectron spray ion source employing a top 5 collision-induced dissociation (CID) fragmentation method.

### Database Search and Spectral Annotation

The Mascot search engine (Mascot 2.2.04, Matrix Science, Boston, MA) was used to search the human proteome contained in the Swiss-Prot database (20,279 reviewed protein sequences, as of September 2013) without any enzymatic restriction (required Mascot ion score ≥20; search engine rank: 1). As a dynamic modification oxidized methionine was allowed. The false discovery rate was estimated with the Percolator algorithm (33) and set to 5%. Peptide lengths for HLA class I-eluted peptides were limited from 8 to 12 amino acids (required charge state: 2–3) and for HLA class II-eluted peptides from 9 to 25 amino acids (required charge state: 2–5). Protein inference was disabled, allowing for multiple protein annotations of peptides. HLA class I annotation was performed using SYFPEITHI (34), and NetMHC (vers. 3.4) (35).

### Whole Transcriptome Sequencing (WTS) and Data Analysis

Whole transcriptome sequencing (WTS) was performed after isolation of mRNA from the patient's tissue samples (mCRC vs. NML) using 100 ng of total RNA and the TruSeq Stranded mRNA Kit (Illumina, San Diego, CA) with 14 cycles of PCR. Tissue sample from patient IRISS06 were processed using 40 ng of total RNA and the TruSeq RNA Access Kit (Illumina) with 15 cycles of amplification. All samples were sequenced on a HiSeq 2500 device (Illumina) as paired-end sequencing. Sequencing depth was 20–40 million cluster/ sample with 68 cycles per read.

#### Data Preprocessing

Adapters were trimmed using SeqPurge [v. 0.1 (36), https://github.com/marc-sturm/ngs-bits]. Trimmed reads were mapped to hg19 using STAR (v. 2.4.2a). Duplicates were removed by picard tools (MarkDuplicates v. 1.85, http://broadinstitute.github.io/picard/).

Expression Analysis: Read counts were calculated using the HTSeq count based method implemented in STAR and Ensembl gene annotations (GRCh37 v. 75). Read counts were normalized using CPM (counts per million mapped reads) and log_2_ fold-changes (FC) were calculated to filter genes with high expression differences.

Variant Calling: Strelka (v. 1.0.11; in matched tumor/normal mode) was used for variant calling and called variants were annotated based on several different databases including among others dbSNP, ExAC, COSMIC, ClinVar and HGMD. SNPeff, Sift, MetaLR, and Polyphen were used to predict effects on gene function. For detection of gene fusions deFuse (v. 0.6.1) was used.

### Peptide Selection

#### Selection of HLA Class I-Restricted Tumor-Specific Peptide Candidates

**Figure 2:**
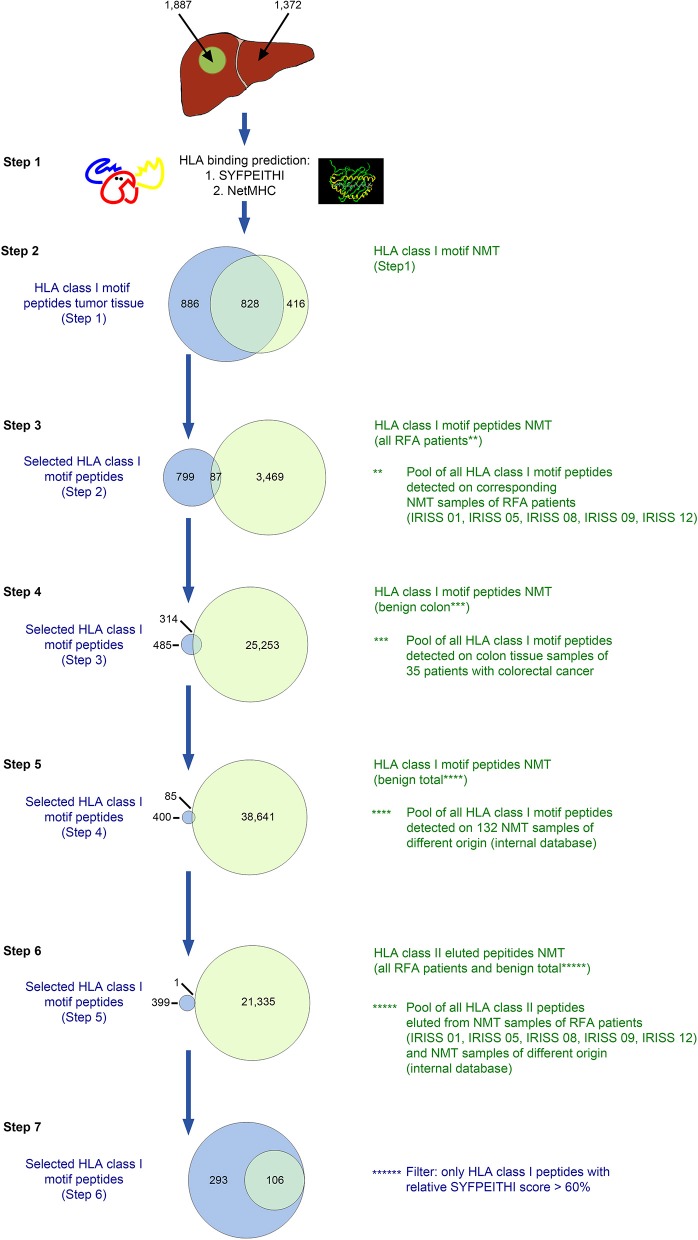
*In silico* selection strategy for candidate HLA class I-presented antigens (exemplified for patient IRISS12). HLA class I-restricted peptides were eluted from mCRC tissue (*n* = 1,887) and corresponding non-malignant liver (NML) tissue (*n* = 1,372) by HLA immunoprecipitation using suitable antibodies followed by uHPLC tandem mass spectrometry (MS/MS). Spectra were annotated using the MASCOT search engine. All peptides eluted were evaluated for their HLA binding affinity using SYFPEITHI and NetMHC version 4.0 (step 1, *n* = 1,714 and *n*=1,244 from malignant and non-malignant tissues, respectively). For further selection, only peptides were included with appropriate HLA class I binding motifs (step 2, *n* = 886). In the following step, the peptides were excluded that were also present on corresponding NML in all included RFA patients (step 3, *n* = 799), 35 non-malignant colon tissues (NMT; step 4, *n* = 485) or on any of 132 non-malignant tissues of different origins (step 5, *n* = 400), as previously reported in Löffler et al. (32). Peptides were further cross-matched with all HLA class II-restricted peptides eluted from NML samples from all included RFA patients (step 6, *n* = 399). As a final step, only peptides which exhibited a SYFPEITHI binding score >60% of the respective maximal allelic score were considered suitable candidate antigens for further manual curation (step 7, *n* = 293). Specific data for all analyzed samples are provided in Supplementary Table 3.

For the six patients of the RFA + surgery group, the multi-step selection approach used included the reassessment of MS/MS detected HLA class I-eluted peptides (a representative example for this approach is provided in Figure 2 for patient IRISS12) regarding their HLA binding affinity by dedicated software [SYFPEITHI >50% max. score (34) and NetMHC v. 3.4 (IC50 <500 nM) (37)]—step 1 (see binders in Table 1 and counts in Supplementary Table 3), subtraction of HLA ligands eluted from non-malignant liver tissue (NML) from those of corresponding mCRC tissue—step 2, as well as the subtraction of all HLA ligands identified on all available non-malignant colon tissue (NMT) samples from the mCRC cohort (32)—step 3. Since the target pool remained extensive at this stage, the strategy was extended to filter out HLA-eluted peptides from non-malignant colon samples available from previous studies—step 4, and subsequently expanded to all HLA class I ligands included in an in-house database comprising 132 non-malignant human tissues from different organs, as already used previously in CRC (32)—step 5. To avoid the selection of peptides presented on HLA class II, any HLA class I-eluted peptides presumably representing shorter length variants of longer HLA class II ligands were discarded—step 6. Finally, to enhance the stringency of selection, HLA class I-eluted peptides were only retained when surpassing a relative SYFPEITHI score of >60% of the maximal allelic score—step 7. For patient IRISS06, step 2 was omitted, because autologous NML tissue was not available. In addition, for patient IRISS09, the HLA-peptide elution resulted unsuccessful for tumor tissue.

#### Selection of HLA Class II-Restricted Tumor-Specific Peptide Candidates

**Figure 3:**
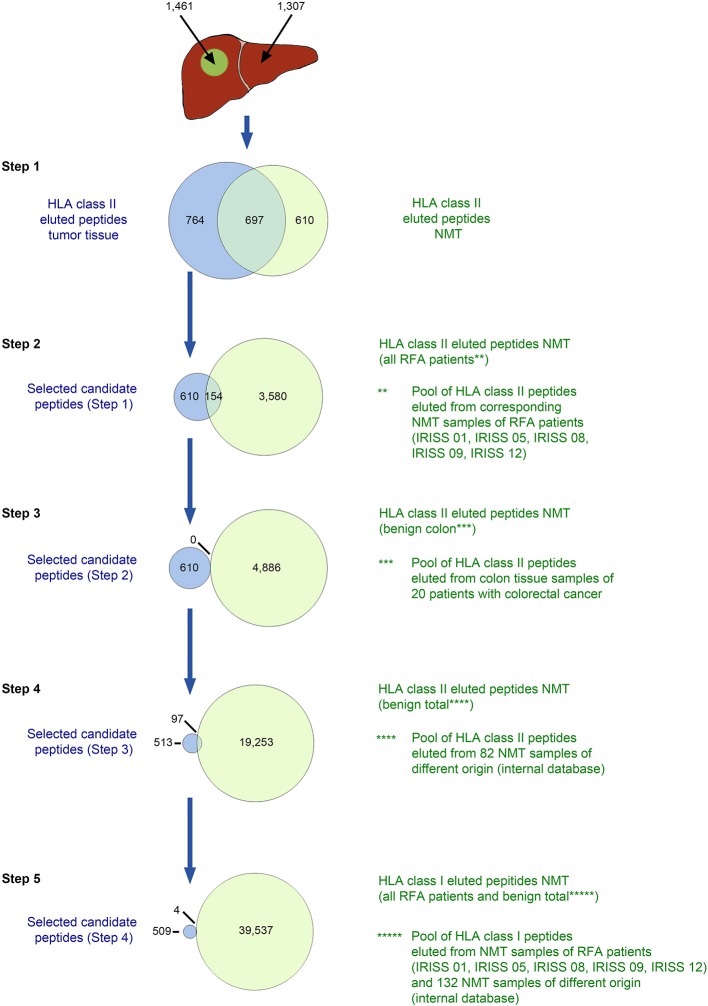
*in silico* selection strategy for candidate HLA class II-presented antigens (exemplified for patient IRISS12). HLA class II-restricted peptides were eluted from mCRC tissue (*n* = 1,461) and corresponding non-malignant liver (NML) tissue (*n* = 1,307) by HLA immunoprecipitation using suitable antibodies followed by tandem mass spectrometry (MS/MS). HLA-eluted peptides were compared between corresponding mCRC and autologous NML tissue and only peptides exclusively found on mCRC were included (step 1, *n* = 764). Further, peptides which were presented on NML of any of the other RFA patients were excluded (step 2, remaining peptides *n* = 610). In the next step, cross-evaluation with a database of 20 non-malignant colon tissues (NMT) (32) could not restrict peptides further (step 3, *n* = 610). Peptides were additionally compared to peptides eluted from 82 non-malignant tissue samples of different origins (32) (step 4, *n* = 513). Before manual assessment, further peptides were excluded when presented as HLA class I antigens on any NML of all RFA patients and 132 tissues of different origins (step 5, *n* = 509). Specific data for all analyzed samples are provided in Supplementary Table 4.

For HLA class II (Figure 3, Supplementary Table 4), peptides eluted from mCRC were initially compared to the peptides characterized by MS/MS on corresponding NML, discarding the overlap—step 1. Subsequently, all HLA class II-eluted peptides of NMT of the entire mCRC cohort were deducted—step 2, as well as all HLA class II-eluted peptides detected in non-malignant colon tissue from previous studies—step 3, then all HLA class II ligands included in an in-house HLA class II peptide database comprising 82 non-malignant human tissues from different organs (32) were eliminated—step 4. Finally, for stringency, all HLA class I peptides comprised in a comprehensive database of benign tissues (*n* = 132) and in the NML tissue from the mCRC cohort were subtracted—step 5. A representative HLA class II selection approach (for patient IRISS12) is provided in Figure 3. For patient IRISS06, step 1 was omitted because autologous NML tissue was not available. Again, for patient IRISS09, the HLA-peptide elution remained unsuccessful for mCRC.

#### Selection of (Predicted) Mutated HLA Ligands

For prediction of mutation-derived HLA ligands, only non-synonymous somatic variants [single nucleotide variants (SNVs) and Insertion/Deletions (InDels)] were selected, when being sequenced with >25 reads in mCRC and simultaneously remaining undetectable in corresponding NML. Additionally, all ambiguous gene transcripts mapping to more than one genetic locus were discarded. Gene fusions were chosen in case >10 split-reads were detectable in mCRC with a probability value >0.8%. Only known driver mutations and variants affecting genes with established relevance for malignant development were selected. For gene fusions the latter was required for at least one of the involved genes.

Non-synonymous somatic variants and gene fusions were translated into the corresponding protein containing the amino acid altered by mutation. The protein sequence flanking the altered amino acid sequence was then disaggregated and screened for HLA class I peptide sequences with a SYFPEITHI score >60% of the maximal allelic score. Mutation containing peptides predicted to bind to the respective patient's HLA class I alleles were extended at the N- and C-terminus to produce a 15 mer peptide, covering both the predicted binding HLA class I peptide sequence as well as peptide sequences showing HLA class II binding properties. Finally, two predicted potential mutated neoantigens were selected, a mutated sequence in the ERRB3 protein for IRISS06 (mERBB3) and a fusion-derived peptide between the two proteins Malic enzyme 2 and SMAD family member 4 (MAOM-SMADA4) for patient IRISS12.

None of the predicted mutation-derived HLA ligands could be confirmed in MS/MS data of HLA ligands eluted from respective mCRC tissue.

#### Selection of Candidate Peptides for Immunological Analyses

Candidate tumor antigen-derived peptides were collated and manually curated for each patient, selecting a manageable set of short and/or long peptides for immunological testing. Criteria for non-mutated peptide selection included increased expression of the source antigen in the tumor as compared to autologous normal tissue [fold change (log_2_); FC], frequency of identification among RFA and CRC (32) cohorts (for HLA class II ligands, length variants were considered), tumor association (e.g., involvement of the source protein in cancerogenesis according to the literature, representation in tumor-associated pathways…). Representation in cancer-associated pathways (Supplementary Table 2) was established by literature research (www.pubmed.gov), as well as through the human protein atlas (www.proteinatlas.org).

The two mentioned predicted mutated peptides were prioritized. Altogether, a ranking list of peptides to be tested was established for each individual patient, and the final number of peptides tested was adjusted to the numbers of available PBMCs (between 6 and 9 peptides/patient, Supplementary Table 2).

### Peptide and HLA-Peptide Monomer Synthesis

Peptides required for T cell stimulation assays (Supplementary Table 2) were synthesized in house (Department of Immunology, University of Tübingen, Germany) by solid-phase synthesis with the 9-fluorenylmethyl-oxycarbonyl/tert-butyl (Fmoc/tBu) strategy (38) in an automated peptide synthesizer (EPS 221, Abimed; ABI 433A, Applied Biosystems). Lyophilized peptides were diluted at 1 mg/ml in distilled water with 10% DMSO and stored at −80°C.

### *In vitro* Stimulation of T Cells and Functional Assays

PBMCs from six patients (RFA + surgery group) were thawed, washed and seeded at ~3–6 × 10^6^ cells per well in a 48-well-plate in IMDM (Lonza, Verviers, Belgium) with 10% heat-inactivated human serum containing 1% penicillin/streptomycin (Sigma-Aldrich, St. Louis, MO) and 50 μM β-mercaptoethanol (Roth, Karlsruhe, Germany) (culture medium). After overnight resting, pooled synthetic peptides were added at 2.5 or 5 μg/ml, for HLA class I and HLA class II peptide stimulations, respectively. Cell culture was performed for 12 days and medium supplemented with recombinant IL-2 (2 ng/ml, R&D Systems, Minneapolis, MN) on days 3, 5, 7, and 9.

Peptide-specific T cells were quantified by intracellular cytokine staining (ICS) for both CD8^+^ and CD4^+^ cells. Directly after 12-day pre-sensitization, cultivated cells were washed and stimulated with the relevant individual peptides (10 μg/ml; in pools or individually) and pre-incubated for 1 h (37°C; 7.5% CO_2_) in the presence of the monoclonal antibody (mAb) CD107a-FITC (clone H4A3, BD Biosciences, Heidelberg, Germany). Phorbol myristate acetate (PMA) (5 ng/ml) plus ionomycin (1 μM) (both Sigma-Aldrich) served as positive control and 10% DMSO was used as negative control. Subsequently, secretion of intracellularly produced cytokines was prevented by adding GolgiSTOP (BD Biosciences) and Brefeldin A (10 μg/ml, Sigma-Aldrich). After a 12 h stimulation period, cells were washed and stained as previously described (39) with mAbs CD3-BV711 (clone OKT3, Biolegend, San Diego, CA), CD8-PE-Cy7 (clone SFCI21Thy2D3, Beckman Coulter, Brea, CA), CD4-APC-Cy7 (clone RPA-T4, BD Biosciences), anti-IFNγ-BV421 (clone 4S.B3, Biolegend), anti-TNF-BV605 (clone Mab11, Biolegend), anti-IL-2-PE and anti-CD154-APC (clone MQ1-17H12 and clone TRAP1, respectively, both BD Biosciences). LIVE/DEAD® Fixable Aqua Dead Cell Stain Kit (ThermoFisher) was included in the stainings. Samples were acquired on a flow cytometer (LSR Fortessa, BD Biosciences) equipped with the DIVA software and analyzed with FlowJo software (TreeStar, Ashland, OR).

The following gating strategy was applied: time gate (histogram)/singlet cells (FSC-H/FSC-A), living cells (FSC-A/ Live/Dead® Fixable Aqua), lymphocytes (FSC-A/SSC-A), CD3^+^ (FSC-A/CD3) CD4^neg^ and CD8^neg^ cells (CD4/CD8); T cell activation (cytokine production, CD107a and CD154 of CD8^+^/CD4^+^ subsets was assessed within the CD4^neg^ and CD8^neg^ lymphocytes, respectively). Results are expressed as % of marker-positive cells within CD4^+^ or CD8^+^ subsets.

Immune responses were considered positive if (I.) the percentage of cytokine producing cells within the sample was 2-fold above the percentage of cytokine producing cells within the corresponding negative control (10% DMSO; no stimulation, as described above), (II.) the number of cytokine producing cells within the sample was ≥20 cells after subtraction of the number of cytokine producing cells within the corresponding negative control (10% DMSO; no stimulation), and (III.) at least two of the five investigated parameters (IFNγ, TNF, IL-2 cytokine production or CD107a, CD154 upregulation) were positive according to the criteria under (I.) and (II.). All dot-plots were audited.

### Immunohistochemistry

Formalin-fixed, paraffin embedded (FFPE) tissue from all 16 patients of both groups was cut in 3–5 μm-thick sections and stained with haematoxylin and eosin (H&E). Immunohistochemistry was performed by an automated immunostainer (Roche Ventana Medical Systems, Tucson, AZ) according to the manufacturer's instructions for open procedures with slight modifications. Samples were stained with antibodies against CD4 (clone SP35, Zytomed Systems, Berlin, Germany), CD8 (clone C8/144B, DAKO, Glostrup, Denmark), CD14 (clone EPR3653, MEDAC Diagnostika, Wedel, Germany), CD19 (clone LE-CD19, Zytomed), CD45RO (clone UCH-L1, Abcam, Cambridge, UK), CD68 (clone KP1, DAKO), Granzyme B (clone 11F1, Novocastra, Wetzlar, Germany), HLA class I (polyclonal, Santa Cruz Biotechnology, Dallas, TX), HLA-DR, -DP, and –DQ (clone CR3-43, DAKO), HSP70 (clone W27, Santa Cruz Biotechnology), IL-10 (polyclonal, Abcam), and LAMP3 (polyclonal, Sigma-Aldrich). Appropriate positive and negative controls were employed to confirm the adequacy of the staining.

Stained slides were digitalized using a Hamamatsu NanoZoomer (C9600-12) using NDP.scan (v. 2.5.88) and NPD.view (v. 2.6.13) software (all from Hamamatsu Photonics, Hamamatsu City, Japan).

Slides were first counted using automated digital slide analysis. For each marker, five representative high-power fields (HPF) were captured using a 200-fold magnification. The number of positive cells was enumerated and the mean for every case was calculated. CD4, CD8, CD19, and CD68 stainings were evaluated using the CD4Quantifier software, which is part of the CognitionMaster Professional Suite (VMscope GmbH, Germany) (40). CD14, CD45RO, HLA-DR, HSP70, IL-10, HLA class I and LAMP3 were evaluated manually. For CD14 and CD45RO, the mean number of positive cells per five HPF was assessed by manual counting. Concerning HLA-DR, we calculated the percentage of positive tumor cells (41). For HSP70, IL-10, and MHC I, we used the immunoreactive score (IRS) (42). In brief, the IRS is calculated by multiplying the number of positive cells (0 = 0%, 1 = 1–10%, 2 = 11–50%, 3 = 51–80%, 4 = >80%) with the staining intensity (0 = no staining, 1 = weak staining, 2 = moderate staining, 3 = strong staining), resulting in a score ranging from 0 to 12.

Slides were also counted manually using the count tool of Adobe Photoshop (v. CC 2018, Adobe Systems, San José, CA). Areas for manual counting were defined as follows: invasive margins were defined as 500 μm in both directions of the tumor border (inwards/outwards) (43). Counting areas were selected using the hot-spot method (good pathological practice) and were defined as areas with subjective/visually most positive stained cells [3 × 0.2 mm^2^ (radius: 252 μm) for each area] (43).

Both automated and manual counting was performed in a blinded fashion by expert pathologists and group assignment was only unblinded to the evaluating pathologists after completion of statistical evaluation.

### Evaluation of Microsatellite Instability (MSI)

Genomic DNA was extracted from macrodissected paraffin sections using the Maxwell® RSC FFPE Plus DNA Purification Kit and the Maxwell® 16 Instrument (Promega, Madison, WI), according to the manufacturer's instructions. Microsatellite PCR in duplicates was performed using genomic DNA and AmpliTaq Gold DNA Polymerase (ThermoFisher) as well-fluorescent labeled primers (Sigma-Aldrich). For GeneScan analysis PCR products were mixed with sample loading solution (Beckman Coulter). The products were separated by capillary electrophoresis on the GenomeLab GeXP Genetic Analysis System and analyzed by the GenomeLab GeXP software 10.2 (Beckman Coulter).

### Statistical Analyses

Mann Whitney *U*-Tests were performed using GraphPad Prism Version 6.0 (GraphPad Software, San Diego, CA). Kaplan Meyer, COX regression and log rank analyses were performed using SPSS Version 24 (IBM, Armonk, NY). Significance levels were set to *p* < 0.05 and respective values considered as statistically significant.

## Results

### Study Design

We recruited two groups of patients with liver metastasis from colorectal carcinoma (mCRC). One group with mCRC was treated merely with surgical resection for their liver lesions (*n* = 7), and another group received RFA first and subsequently a surgical resection (*n* = 9) for the remaining metastases not treated by RFA (Figure 1, Supplementary Table 1). We obtained blood samples from RFA-treated patients before intervention (i.e., RFA treatment followed by surgery) as well as in the course of clinical follow-up (*n* = 6) to obtain peripheral blood mononuclear cells (PBMCs) for immunomonitoring. Further, we obtained tissue samples, encompassing mCRC as well as NML, enabling mRNA analysis by whole transcriptome sequencing (WTS) and the immunoprecipitation and characterization of naturally presented HLA ligands using tandem mass spectrometry (MS/MS). The mean sample weight was 340 mg (range 24–920 mg), yielding on average 835 peptides identified by MS/MS for mCRC and 1,174 peptides for NML (Table 1). On average >75% of HLA class I-eluted peptides from the included samples showed HLA binding properties as corroborated with dedicated software; RNA integrity (RIN) was >6.5 in all cases, except for NML of IRISS06 (RIN = 3.3), which was processed with a high fidelity kit for this reason, to enable the generation of suitable data. Samples with insufficient yields were excluded from downstream analyses.

### Selection of Individual Candidate Antigens

A key challenge for our study was the choice of relevant candidate antigens for testing of T cell recognition. In principle, RFA resembles a whole cell *in situ* vaccination approach, whereby both the priming of novel target-specific T cells as well as boosting of pre-existing T cell responses may occur. Therefore, an extensive spectrum of potential targets prevails that may comprise tumor-specific targets, such as mutated HLA ligands, but also tumor-associated antigens (TAA), a class of tumor antigens that was already previously tested in this setting (27, 44).

In this study, we aimed at a patient-individual selection strategy for candidate HLA class I and II ligands, including MS/MS detected natural HLA ligands exclusive to each patient's own malignant tumor tissue by incorporating information from HLA ligandome as well as from WTS. The approach was complemented by complementary information available from our in house HLA ligand database, which contains an array of natural HLA ligands presented on various different tumor entities (including CRCs) and benign tissues (*n* = 132 and *n* = 82 for HLA class I and HLA class II ligands, respectively, including NML and NMT).

Hence, we used a comprehensive HLA ligand-based multi-step selection strategy (strategies are described in the Materials and Methods section and visualized exemplarily in Figures 2, 3), which was followed for each patient when feasible, aiming at the identification of natural HLA ligands presented by the individual patient's mCRC, ideally derived from tumor-specific proteins, and including both non-mutated as well as selecting mutated peptides, when available. Of note, upon testing all mCRC included in the study were tested as non-MSI high tumors.

An example of the target selection procedure (patient IRISS12) is presented for HLA class I binding candidate peptides in Figure 2, resulting in a decrease of the initial target peptide pool by 84%. The selection procedure for HLA class II binding candidate peptides for the same patient is provided in Figure 3. Respective data for the other included mCRCs disaggregated according to the described steps is presented for HLA class I and class II in Supplementary Tables 3, 4, respectively. The remaining candidate peptides (ranging between 3 and 293 HLA-eluted peptides) encompassed between 1 and 16% of the initially available peptide pool.

High confidence somatic variants identified by WTS were used to predict mutated HLA ligands, selecting only peptides with the required patient-specific HLA class I binding properties. Peptide sequences were elongated to 15 mers, aiming to increase chances for the verification of CD8^+^ and/ or CD4^+^ mediated T cell responses.

Peptides identified through these different procedures were merged and manually curated individually for each patient, selecting a manageable set of short and/or long peptides for immunological testing (6–9 peptides per patient, Supplementary Table 2). Peptides predicted from gene fusions or mutations were preferentially selected, when available (*n* = 2).

### RFA Induces Tumor-Specific T Cell Reactivity

We were able to detect immune responses against various individually selected candidate peptides involving all patients of our small test cohort (*n* = 6); most of these T cell reactivities were directed at long candidate epitopes (presumably HLA class II-restricted). Preexisting antigen-specific T cell responses were confirmed in 4/6 patients, and one of them was assessed as enhanced after RFA treatment (patient IRISS08, CCND1^198−212^). Additionally, *de novo* priming of tumor-specific T cells was observed in two patients, including one immune response against a mutated peptide. These findings can be most probably ascribed to RFA, since the T cell responses could not be measured before treatment in the respective patients (Table 2).

**Table 2:** Overview of T cell reactivity measured by ICS.

**Patient (UPN)**	**Pre-RFA (d0)**	**Post-RFA**	**Effector cells**
IRISS01	CCND1^198−212^	CCND1^198−212^ (1 M, 4 M) [not enhanced]	CD4^+^
IRISS05	Pool: AREG^93−106^, FN1^1789−1804^, CCND1^198−212^	Pool: AREG^93−106^, FN1^1789−1804^, CCND1^198−212^ (1 M) [not enhanced]	CD4^+^
IRISS06		mERBB3^96−110^ (4 M, 7 M) **[induced]**	CD4^+^
IRISS08	IFI6^106−114^	IFI6^106−114^ (6 M) [not enhanced]	CD8^+^
	CCND1^198−212^	CCND1^198−212^ (1 M, 4 M, 6 M) **[enhanced]**	CD4^+^
IRISS09	GPA33^52−67^	GPA33^52−67^ (12.5 M) [not enhanced]	CD4^+^
IRISS12		FN1^1797−1811^ (1.5 M) **[induced]**	CD4^+^

*AREG, amphiregulin; CCND1, cyclin D1; d0, before RFA; FN1, fibronectin 1; GPA33, Glycoprotein A33; IFI6, interferon alpha inducible protein 6; ICS, intracellular cytokine staining; M, month post-RFA; mERBB, (mutated) human epidermal growth factor receptor 3; RFA, radiofrequency ablation; UPN, uniform patient number*.

**Figure 4:**
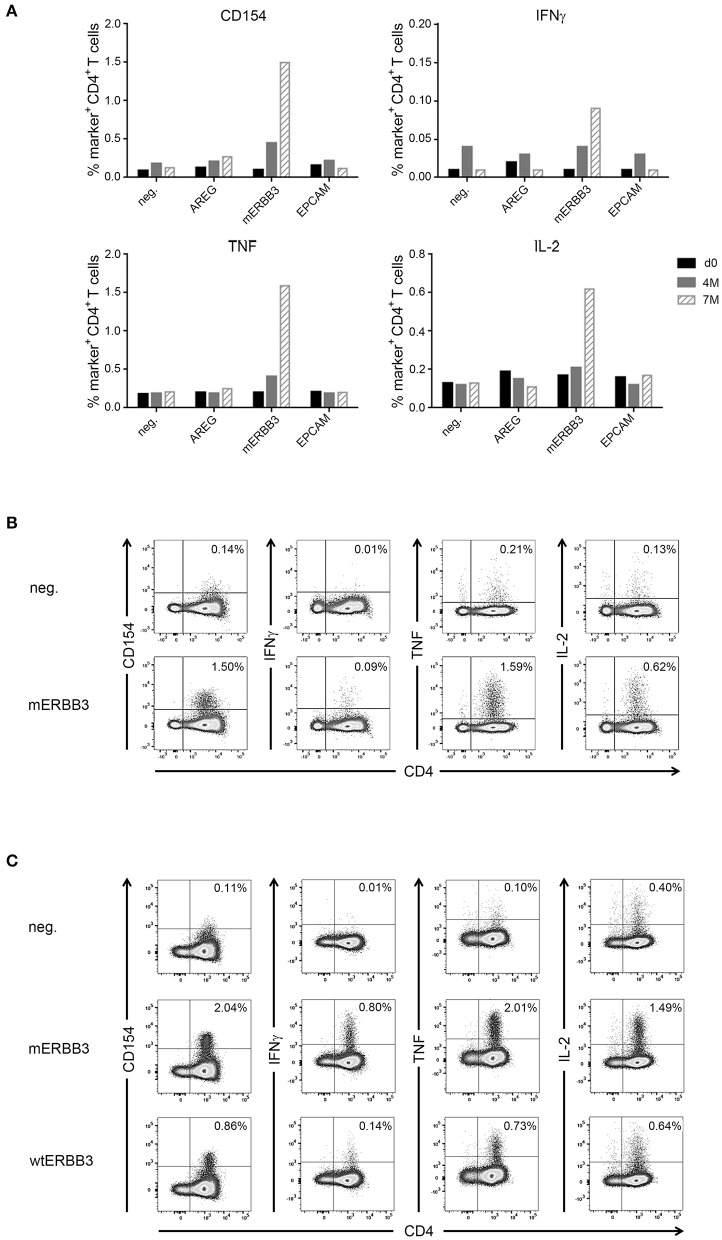
Analysis of antigen- and neoantigen specific CD4^+^ T cells in patient IRISS06. Reactivity of antigen-specific CD4^+^ T cells against selected patient-individual tumor peptides was evaluated by flow cytometry. **(A)** Summary of intracellular cytokine staining (ICS) experiments after 12 day prestimulation followed by restimulation with AREG, mutated ERBB3 (mERBB3) and EpCAM peptides. Patient individual PBMCs obtained before RFA (black bars), as well as 4 months (gray bars) and 7 months (hatched bars) after RFA were assessed. Activation of mERRB3-specific CD4^+^ T cells is reflected by expression of CD154, as well as production of IFNγ, TNF, and IL-2. **(B)** Examples of ICS dot plots (7 month sample) after stimulation with the mERBB3 peptide (TLPLPNLR**L**VRGTQV) after 12 day-prestimulation. Activation of antigen-specific CD4^+^ T cells is reflected by positivity for CD154, as well as cytokine production, including IFNγ, TNF, and IL-2. **(C)** Based on the data presented in **(B)**, a new experiment was performed where PBMCs were tested for reactivity against the mutated and wildtype ERBB3 peptides (TLPLPNLR**L**VRGTQV and TLPLPNLRVVRGTQV, respectively). Activation of CD4^+^ T cells was detected by secretion of IFNγ, TNF, and IL-2, as well as expression of CD154.

In one patient (IRISS06), a CD4^+^ T cell response was induced after RFA, which was directed against one predicted mutated peptide derived from the human epidermal growth factor receptor 3 (mERBB3) containing an amino acid exchange from valine to leucine at position 104 (TLPLPNLR**L**VRGTQV). The induced T cell reactivity to mERBB3 was polyfunctional (Figure 4A; encompassing CD154, interferon γ (IFNγ), tumor necrosis factor (TNF) and interleukin (IL)-2, but not CD107a) with robust responses (~1.4% of the CD4^+^ T cell subset) at 7 months post-RFA treatment (Figure 4B). Importantly, further experiments demonstrated that the corresponding wildtype peptide (wtERBB3: TLPLPNLR**V**VRGTQV) induced strongly attenuated cytokine responses in CD4^+^ T cells, as compared to the mERBB3 peptide (Figure 4C). The other tested peptides (Figure 4A) derived from amphiregulin (AREG) as well as epithelial cell adhesion molecule (EpCAM) did not elicit any detectable T cell responses.

**Figure 5:**
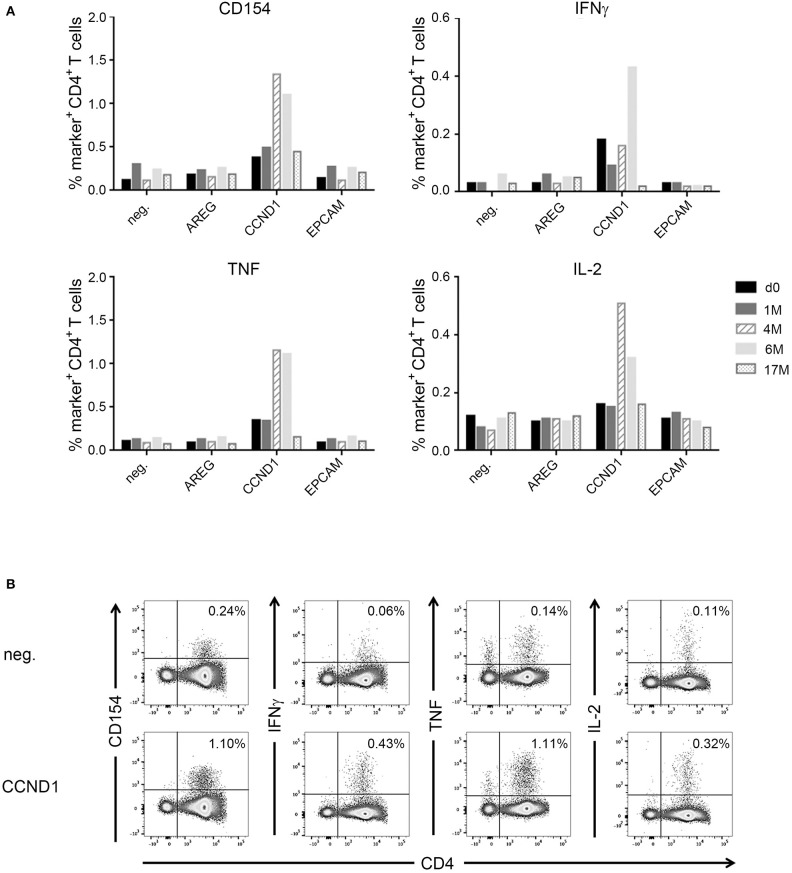
Analysis of antigen-specific CD4^+^ T cells in patient IRISS08. Reactivity of antigen-specific CD4^+^ T cells against selected individual tumor-associated peptides was evaluated by flow cytometry. **(A)** Summary of intracellular cytokine staining (ICS) experiments after 12 day prestimulation followed by restimulation with AREG, CCND1, and EpCAM peptides over time. Patient individual PBMCs obtained before RFA (black bars), as well as 1 month (gray bars), 4 months (hatched bars), 7 months (light gray bars), and 17 months (dotted bars) after RFA were evaluated. Activation of antigen-specific T cells is reflected by expression of CD154, as well as by cytokine production (IFNγ, TNF, and IL-2). **(B)** Examples of ICS dot plots (6 month sample) after stimulation with the HLA class II-restricted CCND1 peptide (NPPSMVAAGSVVAAV) after 12 days prestimulation. Activation of CD4^+^ T cells is reflected by expression of CD154, as well as cytokine production (IFNγ, TNF, and IL-2).

Another patient (IRISS08) showed an enhancement of a pre-existing immune response directed against the long cyclin D1-derived peptide (CCND1) NPPSMVAAGSVVAAV (Supplementary Table 2). This CD4^+^ T cell response was evidenced before as well as 1, 4, and 6 months after RFA with increased functionality after RFA (encompassing positivity for CD154 and cytokines IFNγ, TNF, and IL-2), which peaked at 4 and 6 months but was no longer measurable subsequently (at 17 months post-RFA) (Figures 5A,B). In the same patient, CD8^+^ T cell reactivity against an interferon alpha-inducible protein 6–derived peptide (IFI6: VVIGNIGAL; HLA-A^*^02) was detected before and also after RFA treatment, stimulating IFNγ, TNF and CD107a in ICS, however this response was not boosted (Supplementary Figure 1) and the aggrecan core protein (PGCA)–derived peptide DEFPGVRTY tested simultaneously showed no reactivity.

In addition to these findings, several pre-existing immune responses could be detected, among them CD4^+^ cells responding to the long CCND1-derived peptide previously mentioned, which were not found enhanced after RFA treatment at this time (patient IRISS01) but remained detectable after 1 and 4 months following RFA, encompassing positivity for CD154 as well as positive staining for IFNγ, TNF, IL-2 in ICS (Supplementary Figure 2A). One further patient (IRISS05) was shown to respond to a three peptide pool of long peptides containing the same CCND1-derived peptide NPPSMVAAGSVVAAV as well as a long FN1- (VSVYALKDTLTSRPA) and an AREG-derived peptide (IPGYIVDDSVRVEQ) before as well as 1 month subsequent to RFA with CD4^+^ cells positive for CD107a, CD154 as well as cytokines IFNγ, TNF, and IL-2 (Supplementary Figure 2B). In this case, due to limited sample material, it was impossible to distinguish, which of the peptides was ultimately responsible for the CD4^+^ T cell response. Patient IRISS09 showed a preexisting CD4^+^ T cell response detectable prior to RFA, triggered by the cell surface A33 antigen (GPA33) peptide REGLIQWDKLLLTHTE, which persisted for over 12 months post-RFA (Supplementary Figure 2C). Regrettably, from this patient individual data (transcriptome and HLA ligandome) were lacking, which is why peptides identified in other patients of the study cohort matching to the HLA alleles of interest were selected for evaluation in this case.

Further, we detected an immune response against a long fibronectin peptide (FN1^1797−1811^; patient IRISS12), which proved negative before RFA but showed induction of CD4^+^ cells staining positive for IFNγ, TNF, IL-2 in ICS as well as for CD154, 6 weeks after treatment (Supplementary Figures 2D,E). Whereas, analyses of a predicted peptide derived from a MAOM-SMADA4 fusion (Supplementary Table 2) remained negative.

### Immune Cell Infiltration in Distant Metastases Is Not Increased After RFA

**Figure 6:**
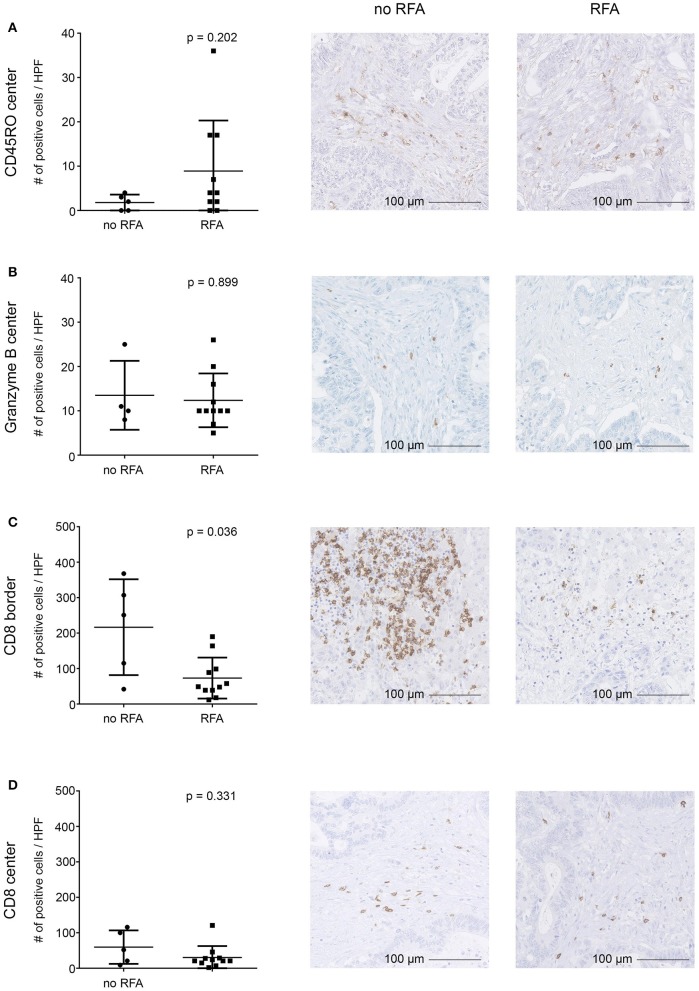
Immunohistochemical evaluation of tumor-infiltrating immune cells into distant CRC liver metastases resected after RFA. Infiltration of immune cells into the tumor center **(A,B,D)** and the invasive tumor margin **(C)** and was assessed by immunohistochemistry revealing comparable infiltration of CD45RO **(A)** and granzyme B **(B)** positive cells, while infiltration of CD8^+^ cells **(C,D)** was diminished in the invasive margin **(C)** but not in the tumor center **(D)** in patients who underwent RFA before surgery as compared to patients who solely underwent surgery. Staining of cells was automatically calculated (left) in digitalized slides. Numbers represent absolute cell counts with specific staining per high power field (HPF) by automated counting. Exemplary immunohistochemistry stainings are provided in the middle (patients after surgical resection) and right (patients after both RFA and surgical resection) columns (20-fold magnification). Differences were assessed using the Mann Whitney *U*-Test with *p* < 0.05 considered as significant.

To determine whether RFA impacts immune cell infiltration into distant, non-ablated, tumor lesions, we assessed our expanded mCRC patent cohort, consisting of patients that received RFA first and a liver resection for additional malignant lesions subsequently (*n* = 9 patients; mCRC lesions were surgically removed ~4 weeks following RFA), as well as a control group of mCRC patients that merely received surgery for their liver metastases (*n* = 7). FFPE tissue was stained by immunohistochemistry for different markers (comprising CD4, CD8, CD14, CD19, CD45RO, CD68, granzyme B, HLA class I, HLA-DR, HLA-DP and HLA–DQ, HSP70, IL-10, and LAMP3). Results were compared between both groups. Overall, no drastic change in the immune cell infiltrate into distant tumor lesions was observed in RFA-pretreated patients, as exemplified by stainings with CD45RO (activated lymphocytes) and granzyme B (cytotoxic lymphocyte effectors) (Figures 6A,B). CD8^+^ cells (potentially cytotoxic T lymphocytes) in the tumor center were generally scarce (<100 cells/HPF) and did not show significant differences between both patient groups neither at the invasive margin nor the tumor center (Figures 6C,D). However, numbers of CD8^+^ cells appeared to be slightly decreased at the invasive margin (Figure 6C). In addition, for patients pre-treated with RFA, we observed significantly decreased numbers of CD4^+^ cells (including cell subsets such as effector T_H_, T_regs_, and possibly also macrophages) both within the tumors and at the invasive margin, when compared to the resection only group (Supplementary Figures 3A,B). Further, HSP70 expression [indicating an inflammatory environment (45)] showed significantly decreased staining in the patients treated with RFA, in contrast to those that only received surgery (Supplementary Figures 3C,D). All other assessed markers, including the expression of HLA molecules, were not found to be significantly different.

Altogether, these findings suggest that no significant influx of immune effector cells was observed 4 weeks after RFA in non-ablated tumor lesions. It should be noted however, that we assessed only lesions that were not treated directly by RFA but distant and resected at later time points (~4 weeks) following RFA treatment.

### Clinical Course of Study Patients

**Figure 7:**
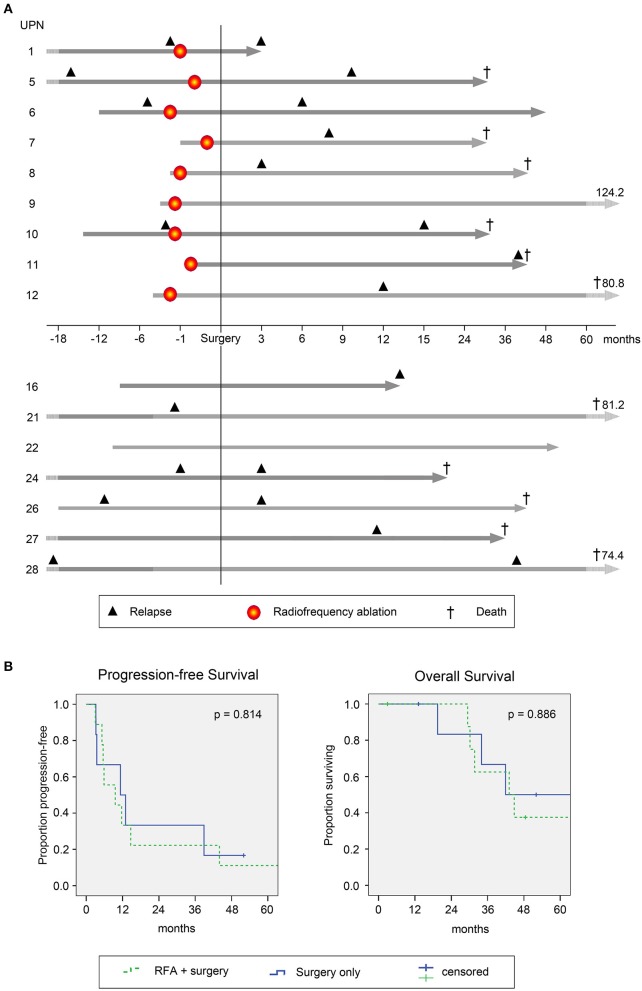
Clinical course and survival of study patients. **(A)** Individual clinical course of patients with colorectal cancer (CRC) metastasized to the liver undergoing RFA followed by surgical resection (top 9 patients, above x-axis) and patients with surgery only (lower 7 patients, below x-axis). Gray arrows indicate time between initial CRC diagnosis and last follow-up. Light gray parts of the arrows indicate variable time spans not fitted to scale. Numbers shown indicate durations of follow-up after surgical resection. In line, respective time spans are normalized to the date of surgery (for comparability with the control group; here defined as day 0). Time points on the x-axis are relative to the time of surgery. Black triangles indicate disease recurrence before (left of y-axis) and after (right of y-axis) study inclusion. Patients with recurrence before RFA and/or surgery represent individuals with metachronous metastasis, while patients without recurrence before RFA and/or surgery had synchronous metastases. Red circles indicate time points of RFA. Crosses indicate passing of patients. **(B)** Progression free (left; PFS) and overall survival (OS) of the complete patient cohort was estimated using Kaplan Meier Regression analysis (*n* = 16). Survival data are presented for patients undergoing RFA followed by surgical resection (green dashed lines, *n* = 9) and for patients with surgical resection only (blue lines, *n* = 7). Differences were assessed by log rank with *p* < 0.05 considered as significant.

For clinical follow-up (data from individual patients are provided in Supplementary Table 1), the date of surgery was defined as day 0 (d0) for both the RFA + surgery and the surgery only (control) groups for reasons of comparability. Patients were followed in median for 43 months (range, 3–124 months). The clinical course of each patient is depicted in Figure 7A. After RFA and surgery, all patients reached complete disease remission (CR) as confirmed by abdominal computed tomography (CT) or magnetic resonance imaging (MRI) scans without signs of active disease.

Median progression free survival (PFS) was 9.6 and 11.3 months for RFA + surgery and surgery only groups, respectively (*p* = 0.814, Figure 7B, left panel). Cumulative incidence of tumor recurrence was 75 and 60% at 12 months for patients undergoing RFA + surgery or surgery only, respectively (*p* = 0.969, hazard ratio 0.978). Sites of recurrence comprised the liver (*n* = 8), the liver and the lung (*n* = 2), as well as the lung, the brain or the abdominal wall and retroperitoneal lymph nodes (*n* = 1 each). Altogether, during follow-up 62% of patients showed tumor recurrence within the liver, whereas in only one of the nine patients treated with RFA (~10%) recurrence was confirmed at the ablation site (for details see Supplementary Table 1).

Upon disease recurrence, patients received standard palliative therapies including repeated local treatment, chemotherapy and best supportive care (BSC), according to local institutional standards. The median overall survival (OS) was comparable for both groups (with 43.1 and 41.9 months in the RFA + surgery vs. surgery only group; *p* = 0.886, Figure 7B, right panel). At the end of follow-up, in the RFA + surgery group three of nine patients remained alive, two with active disease and one in CR. In the surgery only group, two of seven patients remained alive, one with active disease and one in CR. Cause of death was disease recurrence in all cases (*n* = 11).

## Discussion

We and others have previously observed that RFA leads to the induction and release of heat shock proteins (45–47) and is able to induce antigen-specific T cell responses against known tumor-antigens, such as MAGE-A-derived peptides in humans (8, 27). However, so far, these immune responses were only verified at very low frequencies in patients (<5%). Although the patient collective assessed for this study was very limited (*n* = 6), we found T cell responses that were either induced or boosted after RFA (after 1.5–4 months) in 50% of them. Hence, T cell responses were more frequently detected as compared to our previous study (27), which is likely due to the patient-individual strategy of selecting peptides to be assessed as T cell targets. Here we show systemic changes in the immune cell repertoire, encompassing both CD8^+^ and CD4^+^ T cells, responding to long as well as short peptides, fulfilling the characteristics required for HLA presentation.

Using a fully individualized selection strategy, based on patient-specific mCRC HLA ligand profiles as well as whole transcriptome sequencing (WTS), complemented with comprehensive knowledge regarding the HLA ligand repertoire in the context of CRC from previous work (32) and from additional benign and malignant tissues, the broad range of candidate peptides could be substantially minimized for each patient. A multistep selection approach was employed to reduce the amount of candidate peptides to numbers manageable for manual curation. We combined different lines of evidence, including both candidate HLA class I and HLA class II-presented peptides as well as complementary predicted mutated HLA ligands. We thereof selected an individual set of target peptides for each RFA patient for immunological testing. We are aware of the limitations of such an approach that introduces potential –in our view limited– bias, precluding full reproducibility, but it was essential to cope with the challenge of an extensive target pool. Of note, this approach proved effective for the successful identification of targets and for enriching an existing T cell repertoire, validated by the numerous antigen-specific T cell responses evidenced. The obtained results indeed suggest that immunomodulation is a rather frequent feature in the context of RFA, whereas without any obvious clinical effects. These findings are generally in line with reports from previous research in humans, where clinical manifestations of induced immune responses triggered by interventional techniques remain anecdotal (48). This notion is also supported by results from animal testing, where such immune responses are observed but do not appear to be robust or consistent (10). In mouse RFA models for instance, it has been shown that although *in situ* tumor ablation does create a suitable antigen source for generating anti-tumor immunity, the induced T cell responses are usually weak and offer protection from malignancy only in a small subset of animals (11). Of note, in those experiments, performed more than a decade ago, it could already be shown that ICI may potentially augment the occurrence of RFA-induced immune responses.

In our study, mainly non-mutated tumor-antigens were evaluated. The antigenic repertoire of tumor cells comprises a vast array of potential targets, which is partly invisible to confirmatory tools like tandem mass spectrometry (MS/MS), due to specific technical limitations. *In silico*, an excessive quantity of potential HLA-restricted targets can be predicted based on NGS data. Numbers of confirmed HLA ligands are substantially lower than expectable by these predictions, which is likely the reason why MS/MS-confirmed mutated neoantigens remain rather anecdotal at present (18, 19, 49, 50), and suggests that the sole prediction of HLA class I ligands yields an array of false positives (51). Nevertheless, it can be stated that responses to ICI based on tumor mutational burden (TMB) or predicted load of mutated neoantigens may indicate which cancers are more likely to present respective mutated neoantigens on HLA. The reasons for this are indeed multifactorial. We have recently shown that fundamental differences exist between high and low mutated tumors, suggesting this may be of relevance for the probability of presentation of mutated HLA ligands (18). Further, cancer-related pathways may influence the HLA-presented ligandome (32). These alterations may give rise to tumor-specific HLA ligands with wildtype sequence. When sufficiently vetted these targets may prove as a valid alternative to mutations (44, 52) and might further warrant both *in vitro* and *in vivo* investigations as performed in our study.

Descriptions of abscopal effects in mCRC with liver metastasis however are particularly rare even after radiotherapy (53), since the liver is considered inherently tolerogenic and does not favor the induction of immune responses (54).

Furthermore, clinically relevant RFA-induced immunity apparent by distinct clinically recognizable effects in humans is hardly known and most insights in this regard have been derived from animal research. It may be indeed relevant how RFA is precisely performed for the generation of immune responses, since immunological effects may result more effective in malignant tissue that is only treated with subtotal RFA, which has been shown to enable induction of tumor-specific CD8^+^ and CD4^+^ T cells as well as tumor regression in mice (55).

Further, putative influencing factors are *inter alia* the properties of the ablated tumor tissue and numbers and quality of immunogenic epitopes (56). It is easily conceivable that these properties might influence tumor recognition by the immune system, something that has been impressively shown for CRC treated with ICI, where highly mutated cancers responded, whereas sporadic CRCs with low mutation rates did not (21). Here, we observed immune responses to various antigens, among these established tumor-antigens such as cyclin D1 used already in different vaccination approaches (57, 58), but also in one case the recognition of a predicted mutation-derived peptide. The mutation, which was recognized by CD4^+^ T cells, was directed against ERBB3 and could be shown to induce multi-cytokine responses (strongly attenuated for the respective wildtype peptide). Further, this immune response was induced only after RFA and shown as generally increased 7 months after treatment. Of note, a mutation in ERBB2 interacting protein, also recognized by CD4^+^ T cells, exhibiting a T_H_1 profile, has been shown effective for mediating tumor regression in a patient with metastatic cholangiocarcinoma treated by adoptive cell transfer (17).

However, in our study immune infiltrates in non-ablated mCRC liver lesions resected after RFA proved generally scarce by immunohistochemistry. Comparing these non-ablated malignant liver lesions removed after RFA to lesions from mCRC patients with surgery only, significantly lower CD4^+^ cell counts in the tumor center as well as decreased numbers of CD8^+^ and CD4^+^ cells at the tumor border were observed for the RFA + surgery group. These findings support the notion that clinically relevant abscopal effects are rare and not clinically robust. It should be noted though, we do only provide a very limited patient cohort and the analyses only give an impression of the effects observed about 4 weeks after RFA in liver lesions. Further, for instance potential dynamics over time remain unknown. Also, in RFA-treated hepatocellular carcinoma, significantly increased responsiveness to tumor antigens and elevated frequencies of circulating tumor antigen-specific T cells were reported, whereas these effects showed insufficient for tumor control (59). Hence, we may conclude that although immunomodulatory effects in the context of RFA seem to constitute rather the norm than an exception, they may still prove largely ineffective for the induction of robust clinical effects.

Concerning the clinical course of our patients, the combined RFA and surgical treatment, proved comparable to the surgery only group assessed in parallel both with regard to progression free survival (PFS) and overall survival (OS). Some patients in both groups even showed long-term survival. That in mCRC metastasized to the liver, both RFA and surgery and surgical treatment alone may yield similar OS results has recently been concluded from a meta-analysis (60). It is important to realize that patients with several CRC liver metastases are usually considered to be in a palliative stage but may still benefit from a combination of RFA and surgery, as also our survival data suggest. Against this background, larger clinical trials to evaluate the combination of both treatment modalities seem warranted.

In summary, our data show that thermal ablation of metastases induced or boosted tumor-antigen specific T cell responses in half of the mCRC patients evaluated by us. These T cell reactivities can be detected on an individual level, supporting the hypothesis that tumor-directed immunity might include mutated neoantigens and tumor-associated antigens with wildtype sequence that are “selected by nature itself” and that most successful immunotherapies remain limited to strategies strictly confined to individualized approaches (61–64). Since ICI unleash T cell-mediated immune responses non-specifically but rely on natural T cell responses that are individual for each patient (65–67), approaches such as RFA for modulating T cell immunity are anticipated to prove beneficial in this context. There is no doubt to us that T cell responses triggered by thermal ablation generate very limited clinical activity [reviewed in (8)], which is also supported by the data presented in this study. However, our data suggest that RFA-induced immune responses are very frequent and might be boosted by adequate combination treatments. This needs to be investigated in future trials, combining thermal ablation with established (e.g. immune checkpoint inhibitors) and/or novel adjuvants in order to induce more potent –and presumably clinically relevant– immune responses.

## Data Availability Statement

The manuscript datasets, generated, and analyzed during this study have been deposited to the ProteomeXchange Consortium (http://proteomecentral.proteomexchange.org) via the PRoteomics IDEntifications (PRIDE) database partner repository (68) with the dataset identifier PXD015947.

## Ethics Statement

This study was conducted in accordance with the principles of the Declaration of Helsinki and approved by the local institutional review board of the University Hospital Tübingen (Reference No. 169/2005V and 638/2014BO2). All participants provided written informed consent before study inclusion.

## Author Contributions

ML performed *in vitro* experiments, analyzed and interpreted data, wrote the article, supervised the study, and obtained funding. BN performed *in vitro* experiments, analyzed and interpreted data, and wrote the article. DK and LM performed mass spectrometry analyses, analyzed, and interpreted data. GJ and CS performed transcriptome sequencing, analyzed, and interpreted data. PJ, JB, FB, BS, and CD performed analyses of immunohistochemical slides analyzed and interpreted the data. SC, IK, SB, and RL performed patient treatment, managed the clinical part of the study, analyzed, and interpreted the data. SW provided sample material, managed the clinical part of the study, analyzed, and interpreted the data. PP and AK designed the study, performed patient treatment, analyzed, and interpreted the data. TG analyzed and interpreted the data. SS analyzed and interpreted the data and supervised the study. H-GR and CG designed the study, wrote the study protocol, performed *in vitro* experiments, analyzed and interpreted the data, wrote the article, supervised the study, and overall responsibility. SH designed the study, wrote the study protocol, performed *in vitro* experiments, analyzed and interpreted the data, wrote the article, supervised the study, obtained funding, and overall responsibility. All authors revised the article and approved the final version.

### Conflict of Interest

ML, DK, SS, and SH are the inventors of patents owned by Immatics Biotechnologies GmbH. DK is an employee of Immatics biotechnologies GmbH, GJ is employed by CeGaT GmbH, and BN by Roche Diagnostics. H-GR has ownership interest (including patents) in Immatics, CureVac, and Synimmune. The remaining authors declare that the research was conducted in the absence of any commercial or financial relationships that could be construed as a potential conflict of interest.

## Supplementary Material

Supplementary Figure 1 Analysis of antigen-specific CD8^+^ T cells in patient IRISS08. Reactivity of antigen-specific CD8^+^ T cells against selected individual tumor-associated peptides was evaluated by ICS over time before RFA **(A)** and after 6 months **(B)**. T cells were prestimulated for 12 days and restimulated with peptides derived from interferon alpha-inducible protein 6 (IFI6) and aggrecan core protein (PGCA). As positive control, PMA and ionomycin were used. As negative control, 10% DMSO was employed. Positivity criteria used throughout this article are provided in the materials and methods section. Activation of CD8^+^ T cells is reflected by expression of CD107a, as well as cytokine production of IFNγ and TNF.

Supplementary Figure 2 Analysis of antigen-specific T cells in patients IRISS01, IRISS05, IRISS09, and IRISS12. Reactivity of antigen-specific CD4^+^ T cells against selected individual tumor-associated peptides was evaluated by ICS over time. Respective time points of sample obtainment for individual patients are indicated. Patients PBMCs were presensitized for 12 days, restimulated with denoted peptides, and tested in ICS as detailed in Material and Methods. **(A–D)** Expression of CD154, IFN-γ, TNF, and IL-2 in the CD4^+^ subset. For IRISS05 and IRISS12, cell numbers were limited before RFA treatment (day 0). Therefore, peptide pools were used. **(E)** Dot-plots corresponding with tests shown in **(D)** showing FN1-reactive CD4^+^ T cells 1.5M after RFA. Positive responses were defined as detailed in Material and Methods. Additional negative test results are omitted.

Supplementary Figure 3 Immunohistochemical evaluation of CD4 and HSP70 in distant CRC liver metastases resected after RFA. **(A,B)** Infiltration of CD4^+^ cells (including Th, T_regs_, possibly macrophages) into the invasive tumor margin (**A**; border) and tumor center **(B)** was assessed in immunohistochemistry revealing decreased detection of CD4^+^ cells in patients who underwent RFA before surgery. **(C,D)** Heat shock protein 70 (HSP70) expression was significantly diminished in the cytoplasm (cyt., **C**) and in the nucleus (nuc., **D**). Staining of cells was automatically calculated (left) in digitalized slides. Numbers represent absolute cell counts with specific staining per high power field (HPF) by automated counting. Exemplary immunohistochemistry stainings are provided in the middle (patients after surgical resection) and right (patients after both RFA and surgical resection) columns (20-fold magnification). Differences were assessed using the Mann Whitney *U*-Test with *p* < 0.05 considered as significant.

Supplementary Table 1 Patient characteristics.

Supplementary Table 2 Overview of selected individual peptides for immunological testing.

Supplementary Table 3 Selection of HLA class I peptides for identification of potential candidate antigens for immune analyses. A detailed description of the different selection steps can be found in Figure 2 (exemplified for patient IRISS12).

Supplementary Table 4 Selection of HLA class II peptides for identification of potential candidate antigens for immune analyses. A detailed description of the different selection steps can be found in Figure 3 (exemplified for patient IRISS12).

## Abbreviations

APC: allophycocyanin
BSC: best supportive care
BV: brilliant violet
CD: cluster of differentiation
CID: collision-induced dissociation
CPM: counts per million mapped reads
CRC: colorectal cancer
DMSO: dimethylsulfoxide
ERBB3: human epidermal growth factor receptor 3
FCS: fetal calf serum
FFPE, formalin-fixed: paraffin embedded
FN1: fibronectin 1
FSC: forward scatter
H&E: hematoxylin and eosin
HLA: human leucocyte antigen
HSP: heat shock protein
ICI: immune checkpoint inhibition
ICS: intracellular cytokine staining
IFI6: interferon alpha-inducible protein 6
IFN: interferon
IL: interleukin
IRISS, Interventional Radiology, Immunology: Surgery Study
LC: liquid chromatography
LTQ: linear trap quadrupole
mAB: monoclonal antibody
mCRC: metastasized colorectal cancer (to the liver
unless stated otherwise)
mRNA: messenger ribonucleic acid
MHC: major histocompatibility complex
MS: mass spectrometry
MS/MS: tandem mass spectrometry
MSI: microsatellite instable
NML: non-malignant liver
NMT: non-malignant tissue
PGCA: aggrecan core protein
PCR: polymerase chain reaction
PBMC: peripheral blood mononuclear cell
PE: phycoerythrin
PMA: phorbol myristate acetate
RFA: radiofrequency ablation
RNA: ribonucleic acid
TCR: T cell receptor
TNF: tumor necrosis factor
uHPLC: ultra-high-performance liquid chromatography
WTS: whole transcriptome sequencing.
