# Supplementary figures and images for: A Non-interventional Clinical Trial Assessing Immune Responses After Radiofrequency Ablation of Liver Metastases From Colorectal Cancer
Source: Front Immunol. 2019 Nov 19;10:2526. doi: 10.3389/fimmu.2019.02526 (PMC6877671; doi:10.3389/fimmu.2019.02526)

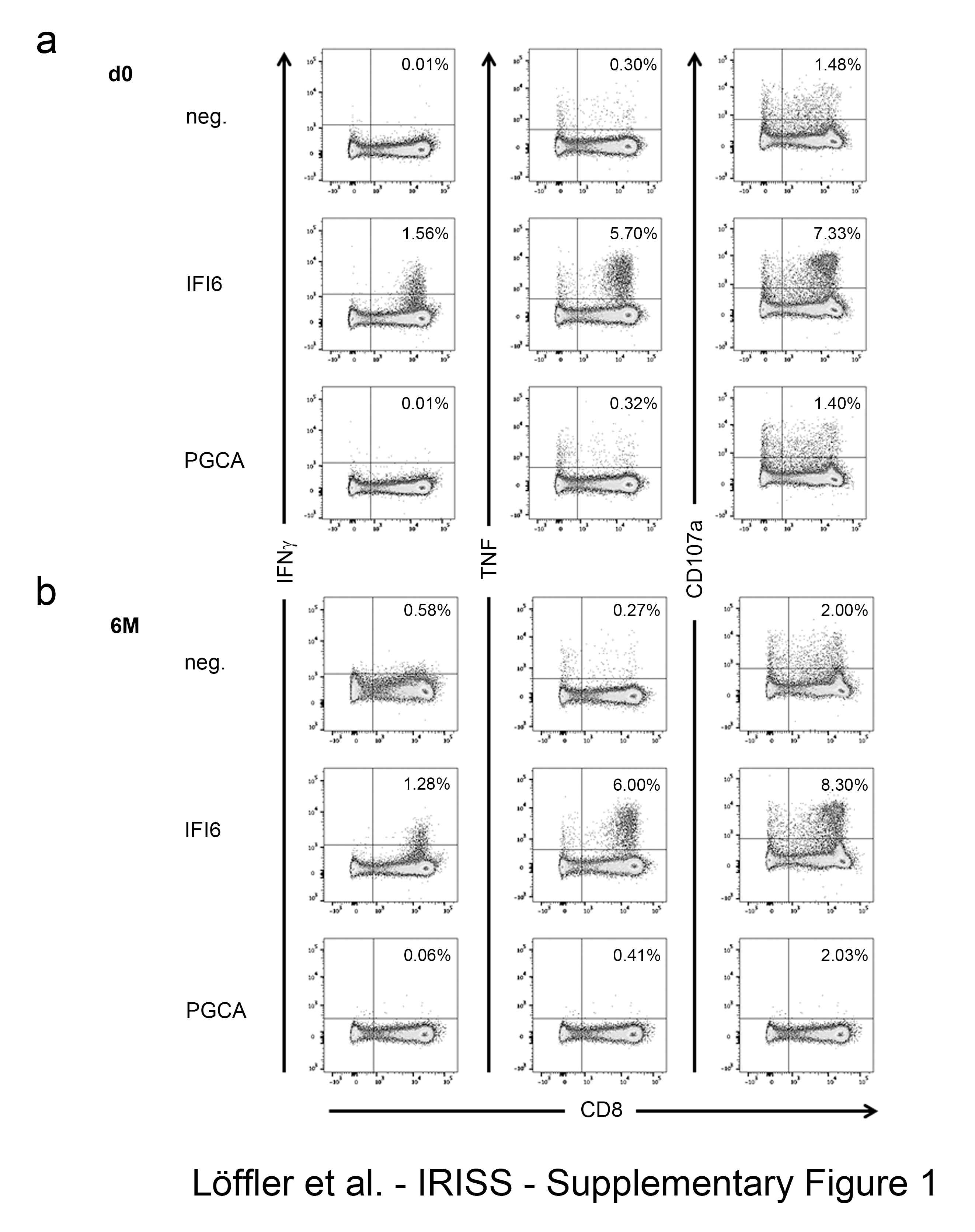

Supplement: Supplementary Figure 1 — Analysis of antigen-specific CD8+ T cells in patient IRISS08. Reactivity of antigen-specific CD8+ T cells against selected individual tumor-associated peptides was evaluated by ICS over time before RFA (A) and after 6 months (B). T cells were prestimulated for 12 days and restimulated with peptides derived from interferon alpha-inducible protein 6 (IFI6) and aggrecan core protein (PGCA). As positive control, PMA and ionomycin were used. As negative control, 10% DMSO was employed. Positivity criteria used throughout this article are provided in the materials and methods section. Activation of CD8+ T cells is reflected by expression of CD107a, as well as cytokine production of IFNγ and TNF. [file Image_1.JPEG]

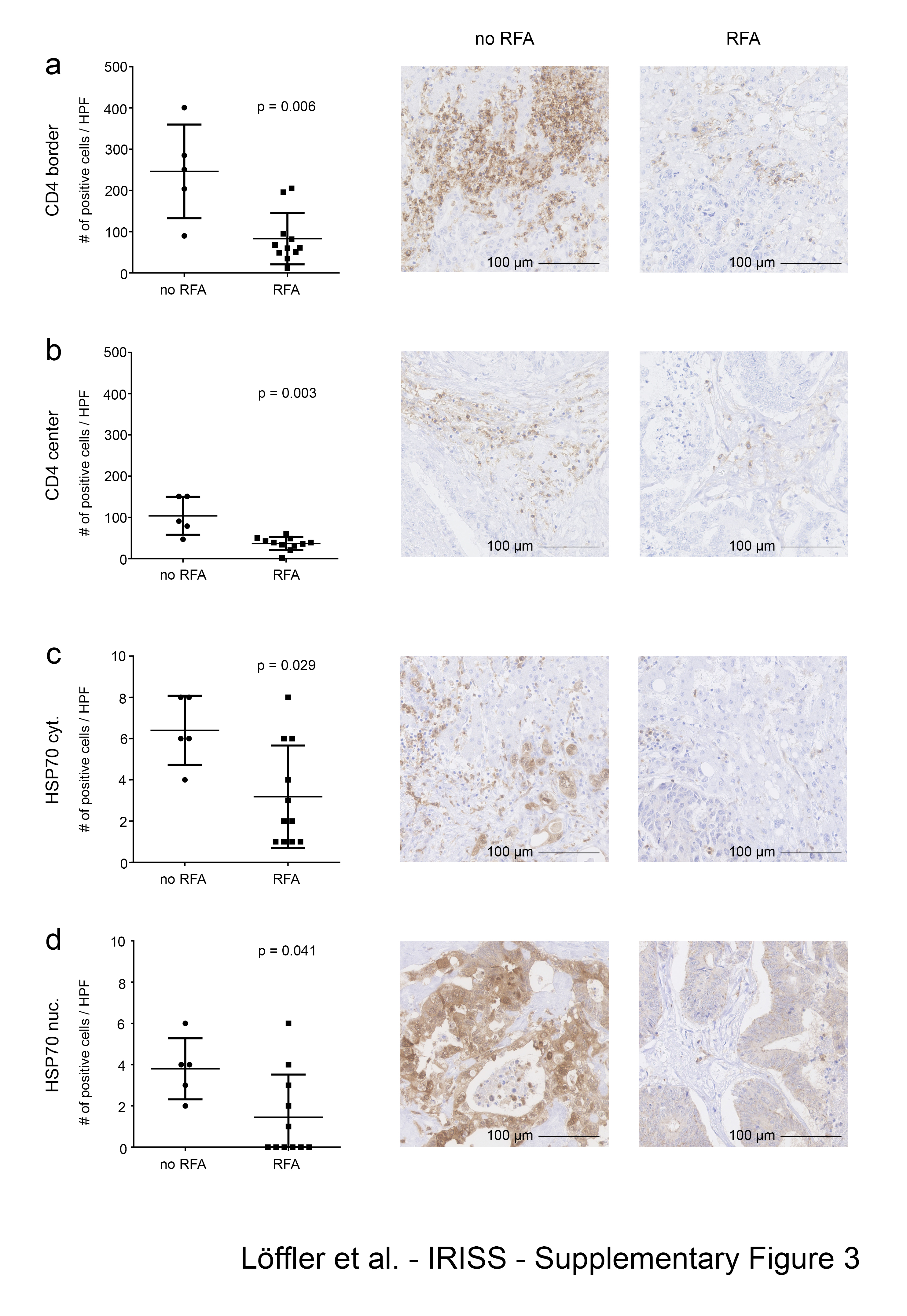

Supplement: Supplementary Figure 3 — Immunohistochemical evaluation of CD4 and HSP70 in distant CRC liver metastases resected after RFA. (A,B) Infiltration of CD4+ cells (including Th, Tregs, possibly macrophages) into the invasive tumor margin (A; border) and tumor center (B) was assessed in immunohistochemistry revealing decreased detection of CD4+ cells in patients who underwent RFA before surgery. (C,D) Heat shock protein 70 (HSP70) expression was significantly diminished in the cytoplasm (cyt., C) and in the nucleus (nuc., D). Staining of cells was automatically calculated (left) in digitalized slides. Numbers represent absolute cell counts with specific staining per high power field (HPF) by automated counting. Exemplary immunohistochemistry stainings are provided in the middle (patients after surgical resection) and right (patients after both RFA and surgical resection) columns (20-fold magnification). Differences were assessed using the Mann Whitney U-Test with p < 0.05 considered as significant. [file Image_3.JPEG]
