# Supplementary material for: A Non-interventional Clinical Trial Assessing Immune Responses After Radiofrequency Ablation of Liver Metastases From Colorectal Cancer
Source: Front Immunol. 2019 Nov 19;10:2526. doi: 10.3389/fimmu.2019.02526 (PMC6877671; doi:10.3389/fimmu.2019.02526)
Supplement: Supplementary Table 1 — Patient characteristics. [file Table_1.pdf]

Supplementary Table 1: Patient characteristics

Abbreviations: adeno: adenocarcinoma; Chx: chemotherapy; CR: complete remission; CRC: colorectal cancer; f: female; HLA: human leukocyte antigen; ID: initial diagnosis; LFU: last follow up; m: male; met.: metastasis; mo: months; MSI: microsatellite instable; MSS: microsatellite stable; n.a.: not applicable; n.d.: no data; OP: operation; RChx: radiochemotherapy; RFA: radiofrequency ablation; RTx: radiotherapy; Seg.: segment; SRG: surgery; TTR: time to relapse; w&w: watch and wait; UIICC: Union internationale contre le cancer.

Patients treated in the RFA + surgery group are shaded in light grey.

[1] age is provided at initial diagnosis.

Patient IRISS #	Group	Sex	Age[1]	Diagnosis	MSI	initial					Therapy before study inclusion	Study inclusion	# of liver lesions	Max. Diameter (RFA / surgery) (Liver Seg.) [mm]	ID to SRG [mo]	RFA to SRG [days]	TTR [mo]	Site of relapse	Re-currence at ablation site	Later Therapy	LFU [mo]	Status at LFU
						T	N	M	G	UICC Stage (VIII)		UICC Stage (VIII)										
**01**	**RFA + Surgery**	m	57	CRC, adeno	low	3	2	1	3	IV	Hemicolectomy<br>Chx<br>Hemihepatectomy	IV	3	RFA: 23x23 (IV)<br>Surgery: 23x24 (I) & 20x19 (II)	19.8	34	3.0	Liver	0	Chx	3.0	Relapse
**05**	**RFA + Surgery**	m	69	CRC, adeno	MSS	3	1	1	2	IV	Hemicolectomy<br>RChx	IV	2	RFA: 18x9 (V)<br>Surgery: 25x17 (VII)	32.6	22	9.6	Brain	0	n.d.	30.2	Death
**06**	**RFA + Surgery**	m	66	CRC, adeno	MSS	3	0	1	2	IV	Hemicolectomy<br>Chx<br>Chemo-embolisation	IV	3	RFA: 31x31 (IV)<br>Surgery: 32x25 (V) & 44x43 (VII)	12.5	54	5.6	Liver	0	RFA<br>Chx	48.5	Relapse
**07**	**RFA + Surgery**	m	47	CRC, adeno	MSS	3	2	1	3	IV	Proctocolectomy	IV	2	RFA: 25x25 (IV)<br>Surgery: 95x92 (IVa/VIII)	1.4	10	5.9	Abdominal wall<br>Lymph nodes	0	RTx<br>surgery	29.4	Relapse
**08**	**RFA + Surgery**	m	61	CRC, adeno	MSS	3	2	1	3	IV	Rectumresection	IV	2	RFA: 13x11 (IVa)<br>Surgery: 60x40 (VII)	1.9	30	3.2	Liver	0	RFA<br>Chx	43.1	Relapse
**09**	**RFA + Surgery**	m	77	CRC, adeno	n.d.	3	1	1	2	IV	Hemicolectomy	IV	2	RFA: 28x22 (VII)<br>Surgery: 65x45 (VI)	2.9	38	n.a.	n.a.	0	n.a.	124.2	CR
**10**	**RFA + Surgery**	m	45	CRC, adeno	MSS	2	1	0	2	IIIA	Rectumresection<br>RChx	IV	3	RFA: 17x18 (VI)<br>Surgery: 14x10 (I) & 13x13 (IVa)	13.5	29	14.7	Liver	0	Chx	31.7	Death
**11**	**RFA + Surgery**	m	77	CRC, addeno	MSS	3	0	1	2	IV	Sigmaresection	IV	2	RFA: 12x12 (VIII)<br>Surgery: 25x17 (II)	2.0	21	44.0	Liver, Lung	0	n.d.	44.8	Relapse
**12**	**RFA + Surgery**	m	79	CRC, adeno	low	3	0	1	2	IV	Hemicolectomy	IV	3	RFA: 19x12 (IVa)<br>Surgery: 23x23 (VI) & 21x21 (VII)	4.2	49	11.7	Liver	1	RFA<br>RFA<br>Chx	80.8	Death

Patient IRISS #	Group	Sex	Age[1]	Diagnosis	MSI	initial						Study inclusion	# of liver lesions	Max. Diameter (RFA / surgery) (Liver Seg.) [mm]	ID to SRG [mo]	RFA to SRG [days]	TTR [mo]	Site of relapse	Re-currence at ablation site	Later Therapy	LFU [mo]	Status at LFU
						T	N	M	G	UICC Stage (VIII)	Therapy before study inclusion	UICC Stage (VIII)										
**16**	**Surgery**	m	47	CRC, adeno	MSS	3	2	1	2	IV	RTx<br>Rectumresection<br>adjuvant Chx	IV	3	Surgery: 12x11 (VII), 14x14 (VIII) & 16x8 (VIII)	9.1	n.a.	13.0	Liver, Lung	n.a.	n.d.	13.2	Relapse
**21**	**Surgery**	m	53	CRC, adeno	MSS	1	1	1	3	IV	Chx<br>RFA	IV	2	Surgery: 7x7 (VI) & 19x15 (VI)	24.9	n.a.		n.a.	n.a.	n.d.	81.2	n.d.
**22**	**Surgery**	m	60	CRC, adeno	MSS	2	0	1	2	IV	Chx<br>Hemicolectomy	IV	1	Surgery: 21x17 (IVb)	9.7	n.a.	n.a.	n.a.	n.a.	n.a.	52.0	CR
**24**	**Surgery**	m	61	CRC, adeno	MSS	3	1	0	2	IIIB	Hemicolectomy<br>Chx	IV	2	Surgery: 20x21 (VI) & 35x30 (VI/VII)	38.8	n.a.	3.2	Lung	n.a.	w&w	19.5	Relapse
**26**	**Surgery**	m	56	CRC, adeno	n.d.	3	1	0	2	IIIB	Resection<br>Chx	IV	7	Surgery: 18x18 (II), 7x7 (II), 18x18 (II/IV), 10x10 (V), 27x27 (VII), 10x10 (VIII) & 6x6 (VIII)	18.1	n.a.	3.5	Liver	n.a.	Chx<br>RFA	41.9	Relapse
**27**	**Surgery**	f	69	CRC, adeno	MSS	1	0	0	2	I	Hemicolectomy	IV	1	Surgery: 40x35 (IVa)	24.2	n.a.	11.3	Liver	n.a.	Chx<br>surgery	34.0	Death
**28**	**Surgery**	f	62	CRC, adeno	MSS	3	0	0	2	IIA	Hemicolectomy<br>Hemihepatectomy	IV	1	Surgery: 20x14 (II)	101.7	n.a.	38.9	Liver	n.a.	n.d.	74.4	Relapse
