# Supplementary material for: A Non-interventional Clinical Trial Assessing Immune Responses After Radiofrequency Ablation of Liver Metastases From Colorectal Cancer
Source: Front Immunol. 2019 Nov 19;10:2526. doi: 10.3389/fimmu.2019.02526 (PMC6877671; doi:10.3389/fimmu.2019.02526)
Supplement: Supplementary Table 2 — Overview of selected individual peptides for immunological testing. [file Table_2.pdf]

Supplementary Table 2: Overview of selected individual peptides for immunological testing.
<u>Abbreviations:</u> aa: amino acid; FC: fold change; HLA: human leukocyte antigen; ID: identifier; n.a.: not applicable; n.k.: not known.

HLA class I:

						SYFPEITHI		NetMHC								
Sequence	Source protein	UniProt ID	aa position	Mass [Da]	HLA	HLA allele	Relative score [%]	HLA allele	IC_{50} score [nM]	Evaluated in patient (UPN)	FC value (log2)	Frequency in CRC patients	Frequency in RFA patients	Involvement in cancer	Detected in patient	Detected by
TTDCLQILAY	CTNNB1/ PLAK	P35222 / P14923	297-306	1139.55	A*01	A*01:01	87.2	A*01:01	10	IRISS12	2.6	0/35	1/5	yes	IRISS12	MS/MS
DTDSAIGSFRY	DUS27	Q5VZP5	888-898	1230.55	A*01	A*01:01	82.1	A*01:01	9	IRISS05<br>IRISS09<br>IRISS12	4.8<br>4.8<br>4.2	2/35	2/5	n.k.	IRISS05<br>IRISS12	MS/MS
PSENNKLTY	VAMP7	P51809	37-45	1964.51	A*01	A*01:01	75.0	A*01:01	22	IRISS09	1.3	3/35	1/5	n.k.	IRISS12	MS/MS
VVIGNIGAL	IFI6	P09912	106-114	854.52	A*02	A*02:01	66.7	A*02:50	48	IRISS08	7.0	0/35	1/5	n.k.	IRISS12	MS/MS
GLALKVQEV	TENS4	Q8IZW8	484-493	955.57	A*02	A*02:01	83.3	A*02:50	2	IRISS05<br>IRISS08	< 0<br>< 0	9/35	1/5	yes	IRISS08	MS/MS
KYISGPHEL	CENPF	P49454	1271-1279	1042.54	A*24	A*24:02	77.4	A*24:03	6	IRISS01	4.2	2/35	0/5	yes		MS/MS
IYFEYSHAF	MSH3	P20585	461-469	1175.53	A*24	A*24:02	71.0	A*24:03	6	IRISS06	2.5	0/35	1/5	yes	IRISS06	MS/MS
VYFSKSEQL	EPHA2	P29317	574-582	1099.56	A*24	A*24:02	77.4	A*24:03	11	IRISS01	8.2	0/35	0/5	yes		MS/MS
NYMPGQLTI	MACC1	Q6ZN28	394-402	1035.51	A*24	A*24:02	67.7	A*24:03	6	IRISS01	1.8	1/35	0/5	yes		MS/MS
YYQDTPKQI	NOP16	Q9Y3C1	140-148	1154.56	A*24	A*24:02	71.0	A*24:03	7	IRISS01	2.0	2/35	0/5	yes		MS/MS
VLKGKKLSL	ERD21 / ERD22	P24390 / P33947	202-210	984.67	B*08	B*08:01	86.1	A*02:50	2	IRISS09	1.6	3/35	1/5	n.k.	IRISS12	MS/MS
VPKEKDEM	KDM1A	O60341	370-377	974.47	B*08	B*08:01	72.5	B*08:01	1756	IRISS12	7.4	0/35	1/5	n.k.	IRISS12	MS/MS
TQRGALFLLF	SRPX2	Q60687	6-15	1164.67	B*15	B*15:01	63.6	B*15:03	4	IRISS06	1.8	2/53	1/5	yes		MS/MS
QQMVVAHQY	TENS3	Q68CZ2	593-601	1102.52	B*15	B*15:01	81.5	B*15:03	22	IRISS06	2.0	0/35	0/5	n.k.		MS/MS
DEFPGVRTY	PGCA	P16112	225-233	1082.50	B*18	B*18:01	78.8	B*18:01	3	IRISS05<br>IRISS08	0.4<br>0.4	1/35	2/5	n.k.	IRISS05<br>IRISS08	MS/MS
IRPNIILVL	SULF1 / SULF2	Q8IWU6 / Q8IWU5	41-49	1049.7	B*27	B*27:05	71.1	B*27:20	25	IRISS12	1.3	9/35	0/5	yes		MS/MS
AEIEADRSY	LAMC2	Q13753	837-845	1053.48	B*44	B*44:02	73.0	B*44:03	8	IRISS01	4.5	2/35	0/5	yes		MS/MS

HLA class II:

Sequence	Source protein	UniProt ID	aa position	Mass [Da]	HLA	Evaluated in patient (UPN)	FC value (log2)	Frequency in CRC patients	Frequency in RFA patients	Involvement in cancer	Detected in auto-logous tumor tissue	Detected by
IPGYIVDDSVRVEQ	AREG	P15514	93-106	1588.81	DR	IRISS05<br>IRISS06<br>IRISS08<br>IRISS09<br>IRISS12	2.7<br>2.7<br>2.7<br>2.2<br>2.2	3/19	1/4	yes	IRISS12 (several length variants)	MS/MS
NPPAQYSWLIDGNIQQH	CEAM5	P06731	442-458	2209.06	DR	IRISS01	< 0	0/19	1/4	yes	IRISS12 (several length variants)	MS/MS
REGLIQWDKLLLTHTE	GPA33	Q99795	52-67	1951.05	DR	IRISS01<br>IRISS09	4.7<br>4.7	0/19	1/4	yes	IRISS01 (several length variants)	MS/MS
YELQYYEKE	EPHB2	P29323	469-477	1263.57	DR	IRISS12	7.4	0/19	14	yes	IRISS12 (several length variants)	MS/MS
DPKFITSILYENNVITI	EPCAM	P16422	177-193	1979.06	DR	IRISS01<br>IRISS06<br>IRISS08<br>IRISS09	3.7<br>3.9<br>3.9<br>3.7	0/19	1/4	yes	IRISS01 (several length variants)	MS/MS
VSVYALKDTLTSRPA	FN1	P02751	1789-1804	1619.89	DR	IRISS05	3.4	0/19	0/4	yes		MS/MS
DTLTSRPAQGVVTTL	FN1	P02751	1797-1811	1343.70	DR	IRISS09<br>IRISS12	2.8	0/19	1/4	yes	IRISS12	MS/MS
NPPSMVAAGSVVAAV	CCND1	P24385	198-212	1254.66	DR	IRISS01<br>IRISS05<br>IRISS08	0.9<br>1.1<br>1.2	1/19	3/4	yes	IRISS01<br>IRISS05<br>IRISS08	MS/MS
VYIF**PG**QPASILGGS (Gene fusion)	MAOM-SMAD4	P23368 / Q13485	468-473; 224-232	1504.79	DR	IRISS12	< 0	n.a.	1/4	yes	IRISS12 (fusion)	WTS predicted
TLPLPNLRLVRGTQV (Mutation V104L)	mERBB3	P21860	96-110	1676.01	DR	IRISS06	n.a.	n.a.	1/4	yes	IRISS06 mutation	WTS predicted
TLPLPNLRVVRGTQV (Wildtype)	ERBB3	P21860	96-110	1661.99	DR	IRISS06	n.a.	n.a.	n.a.	yes	corres-ponding wildtype peptide	MS/MS
