# Supplementary material for: A Non-interventional Clinical Trial Assessing Immune Responses After Radiofrequency Ablation of Liver Metastases From Colorectal Cancer
Source: Front Immunol. 2019 Nov 19;10:2526. doi: 10.3389/fimmu.2019.02526 (PMC6877671; doi:10.3389/fimmu.2019.02526)
Supplement: Supplementary Table 3 — Selection of HLA class I peptides for identification of potential candidate antigens for immune analyses. A detailed description of the different selection steps can be found in Figure 2 (exemplified for patient IRISS12). [file Table_3.pdf]

Supplementary Table 3: Selection of HLA class I peptides for identification of potential candidate antigens for immune analyses.

A detailed description of the different selection steps can be found in figure 2 (exemplified for patient IRISS 12).

	**HLA class I peptides**								
**UPN**	**Step 0**	**Step 1**	**Step 2**	**Step 3**	**Step 4**	**Step 5**	**Step 6**	**Step 7**	**Selection rate [%]**
**IRISS 01**	1785	1508	552	521	260	237	237	181	10
**IRISS 05**	260	198	25	27	8	7	5	3	1
**IRISS 06**	711	666	n.d.	438	69	46	44	33	5
**IRISS 08**	231	175	26	17	10	9	9	2	1
**IRISS 09**	136	n.d.	n.d.	n.d.	n.d.	n.d.	n.d.	n.d.	n.d.
**IRISS 12**	1887	1714	886	799	485	400	399	293	16

<u>Abbreviations:</u>
n.d.: not determined; UPN: uniform patient number.
