# Supplementary material for: A Non-interventional Clinical Trial Assessing Immune Responses After Radiofrequency Ablation of Liver Metastases From Colorectal Cancer
Source: Front Immunol. 2019 Nov 19;10:2526. doi: 10.3389/fimmu.2019.02526 (PMC6877671; doi:10.3389/fimmu.2019.02526)
Supplement: Supplementary Table 4 — Selection of HLA class II peptides for identification of potential candidate antigens for immune analyses. A detailed description of the different selection steps can be found in Figure 3 (exemplified for patient IRISS12). [file Table_4.pdf]

Supplementary Table 4: Selection of HLA class II peptides for identification of potential candidate antigens for immune analyses.

A detailed description of the different selection steps can be found in figure 3 (exemplified for patient IRISS 12).

	HLA class II peptides						
**UPN**	**Step 0**	**Step 1**	**Step 2**	**Step 3**	**Step 4**	**Step 5**	**Selection rate [%]**
**IRISS 01**	850	143	132	117	89	84	10
**IRISS 05**	556	427	394	387	299	284	51
**IRISS 06**	7	n.d.	n.d.	n.d.	n.d.	n.d.	n.d.
**IRISS 08**	220	86	68	57	51	51	23
**IRISS 09**	17	n.d.	n.d.	n.d.	n.d.	n.d.	n.d.
**IRISS 12**	1461	764	610	610	513	509	35

Abbreviations:
n.d.: not determined; UPN: uniform patient number.
